# Supplementary material for: External validation of the QCovid 2 and 3 risk prediction algorithms for risk of COVID-19 hospitalisation and mortality in adults: a national cohort study in Scotland
Source: BMJ Open. 2023 Dec 27;13(12):e075958. doi: 10.1136/bmjopen-2023-075958 (PMC10753764; doi:10.1136/bmjopen-2023-075958)
Supplement: Supplementary data [file bmjopen-2023-075958supp001.pdf]

Table S1: Summary statistics for the full cohort and 10% sample

Characteristics	Levels	Entire cohort	10% cohort sample
Total		4650585 (100.0)	465058 (100.0)
COVID-19 Deaths		4049 (0.1)	393 (0.1)
COVID-19 Hospitalisations		9547 (0.2)	763 (0.2)
Sex	Female	2358990 (50.7)	235821 (50.7)
	Male	2291595 (49.3)	229237 (49.3)
Age	Mean (SD)	50.3 (18.8)	50.3 (18.9)
Age group	19-39	1581219 (34.0)	158052 (34.0)
	40-59	1533605 (33.0)	152985 (32.9)
	60-74	978827 (21.0)	98190 (21.1)
	75-100	556934 (12.0)	55831 (12.0)
SIMD quintile	1 - Most deprived	925925 (19.9)	92546 (19.9)
	2	918884 (19.8)	92073 (19.8)
	3	918091 (19.7)	91868 (19.8)
	4	921838 (19.8)	92219 (19.8)
	5 - Least deprived	924963 (19.9)	92392 (19.9)
	(Missing)	40884 (0.9)	3960 (0.9)
Urban rural classification	Large Urban Areas	1671719 (35.9)	166887 (35.9)
	Other Urban Areas	1652152 (35.5)	165274 (35.5)
	Accessible Small Towns	424031 (9.1)	42739 (9.2)
	Remote Small Towns	221513 (4.8)	22271 (4.8)
	Accessible Rural	413436 (8.9)	41170 (8.9)
	Remote Rural	226850 (4.9)	22757 (4.9)
	(Missing)	40884 (0.9)	3960 (0.9)
Ethnicity	Asian	102490 (2.2)	10250 (2.2)
	Black	25123 (0.5)	2567 (0.6)
	Mixed	21139 (0.5)	2148 (0.5)

	Other	23584 (0.5)	2383 (0.5)
	Unknown	1562888 (33.6)	156408 (33.6)
	White	2915361 (62.7)	291302 (62.6)
Number of risk groups	0	2725853 (58.6)	272930 (58.7)
	1	1249848 (26.9)	125016 (26.9)
	2	434728 (9.3)	43153 (9.3)
	3 - 4	210691 (4.5)	21024 (4.5)
	5+	29465 (0.6)	2935 (0.6)
BMI	15-18.5	48651 (1.0)	4772 (1.0)
	18.5-25	543001 (11.7)	54377 (11.7)
	25-30	674760 (14.5)	67921 (14.6)
	30-35	431788 (9.3)	43170 (9.3)
	35-40	198273 (4.3)	19835 (4.3)
	40+	133917 (2.9)	13226 (2.8)
	(Missing)	2620195 (56.3)	261757 (56.3)
Housing category	Neither	4624584 (99.4)	462533 (99.5)
	Care home	25217 (0.5)	2457 (0.5)
	Homeless	784 (0.0)	68 (0.0)
Learning disability/Down's syndrome	Neither	4600228 (98.9)	460016 (98.9)
	Down's syndrome	1526 (0.0)	144 (0.0)
	Learning disability	48831 (1.0)	4898 (1.1)
Chronic Kidney Disease	No CKD	4484131 (96.4)	448600 (96.5)
	CKD 3	166431 (3.6)	16455 (3.5)
	CKD 4	19 (0.0)	*
	CKD 5	4 (0.0)	*
Atrial Fibrillation		114239 (2.5)	11502 (2.5)
Asthma		494549 (10.6)	49499 (10.6)
Blood cancer		20162 (0.4)	2049 (0.4)

Congestive Cardiac Failure		46961 (1.0)	4748 (1.0)
Cerebral Palsy		5521 (0.1)	529 (0.1)
Coronary heart disease		191035 (4.1)	19132 (4.1)
Liver cirrhosis		10882 (0.2)	1116 (0.2)
Congenital heart disease		18969 (0.4)	1854 (0.4)
COPD		118884 (2.6)	11922 (2.6)
Dementia		23258 (0.5)	2202 (0.5)
Diabetes Type 1		65238 (1.4)	6434 (1.4)
Diabetes Type 2		205608 (4.4)	20446 (4.4)
Epilepsy		4032 (0.1)	386 (0.1)
Hip, wrist, spine, humerus fracture		1638 (0.0)	140 (0.0)
HIV/AIDS		18937 (0.4)	1865 (0.4)
Severe combined immunodeficiency syndrome		9707 (0.2)	987 (0.2)
Neurological conditions		6916 (0.1)	668 (0.1)
Parkinsons disease		23496 (0.5)	2367 (0.5)
Pulmonary hypertension		11937 (0.3)	1212 (0.3)
Cystic fibrosis, bronchiectasis or alveolitis		88090 (1.9)	8852 (1.9)
Peripheral vascular disease		5452 (0.1)	538 (0.1)
SLE or rheumatoid arthritis		19750 (0.4)	1892 (0.4)
Lung, oral cancer		205 (0.0)	8 (0.0)

Severe mental illness		115619 (2.5)	11431 (2.5)
Sickle cell disease or combined immune deficiency syndrome		41948 (0.9)	4198 (0.9)
Stroke, transient ischaemic attack		22733 (0.5)	2309 (0.5)
Venous thromboembolism		265285 (5.7)	26697 (5.7)

Box S1: Predictor variables in the QCovid 3 algorithm

- Vaccine dose
- Age in years (continuous)
- Sex
- Ethnicity in ten categories (Bangladeshi, Black African, Black Caribbean, Chinese, Indian, Mixed, Pakistani, White British, White other, Other, unknown)
- Townsend deprivation score (continuous)
- Body Mass Index (kg/m2)
- Accommodation (Neither homeless nor care home, care home or nursing home)
- Chronic kidney disease (CKD) – (no CKD, CKD3, CKD4, CKD5, unknown)
- Learning disability (No learning disability, Down’s Syndrome, other learning disability)
- Chemotherapy in last 12 months (none, Chemotherapy group A, B, C, unknown)
- Blood Cancer
- Bone Marrow transplant in the past 6 months
- Rare Lung Diseases (cystic fibrosis, bronchiectasis, or alveolitis)
- Respiratory cancer
- Radiotherapy in last 6 months
- Solid organ transplant
- Prescribed immunosuppressant medication by GP
- Prescribed leukotriene or long-acting beta blockers
- Prescribed regular prednisolone
- Sickle cell disease
- Diabetes
- Chronic obstructive pulmonary disease (COPD)
- Asthma
- Rare pulmonary diseases
- Pulmonary hypertension or pulmonary fibrosis
- Coronary heart disease
- Stroke
- Atrial Fibrillation
- Congestive cardiac failure
- Venous thromboembolism
- Peripheral vascular disease
- Congenital heart disease
- Dementia
- Heart Failure
- Parkinson's disease
- Epilepsy
- Rare neurological conditions
- Cerebral palsy
- Severe mental illness (bipolar disorder, schizophrenia, severe depression)
- Osteoporotic fracture
- Rheumatoid arthritis or Systemic lupus erythematosus
- Cirrhosis of the liver
- Inflammatory Bowel Disease
- HIV/AIDS
- Severe Combined Immunodeficiency
- Previous Positive SARS-CoV-2 positive test result
- Background rate of RT-PCR positivity per 100,000 people
