# Supplementary material for: External validation of the QCovid 2 and 3 risk prediction algorithms for risk of COVID-19 hospitalisation and mortality in adults: a national cohort study in Scotland
Source: BMJ Open. 2023 Dec 27;13(12):e075958. doi: 10.1136/bmjopen-2023-075958 (PMC10753764; doi:10.1136/bmjopen-2023-075958)
Supplement: Author's manuscript [file bmjopen-2023-075958.draft_revisions.pdf]

BMJ Open is committed to open peer review. As part of this commitment we make the peer review history of every article we publish publicly available.

When an article is published we post the peer reviewers' comments and the authors' responses online. We also post the versions of the paper that were used during peer review. These are the versions that the peer review comments apply to.

The versions of the paper that follow are the versions that were submitted during the peer review process. They are not the versions of record or the final published versions. They should not be cited or distributed as the published version of this manuscript.

BMJ Open is an open access journal and the full, final, typeset and author-corrected version of record of the manuscript is available on our site with no access controls, subscription charges or pay-per-view fees (http://bmjopen.bmj.com).

If you have any questions on BMJ Open's open peer review process please email info.bmjopen@bmj.com

BMJ Open

External validation of the QCovid 2 and 3 risk prediction algorithms for risk of COVID-19 hospitalisation and mortality in adults: national cohort study in Scotland

Journal:	*BMJ Open*
Manuscript ID	bmjopen-2023-075958
Article Type:	Original research
Date Submitted by the Author:	29-May-2023

Complete List of Authors:	Kerr, Steven; University of Edinburgh<br>Millington, Tristan; The University of Edinburgh Usher Institute of Population Health Sciences and Informatics,<br>Ruden, Igor; The University of Edinburgh<br>McCowan, Colin ; University of St. Andrews<br>Tibble, Holly; University of Edinburgh, Usher Institute of Population Health Sciences and Informatics; Asthma UK Centre for Applied Research,<br>Jeffrey, Karen; University of Edinburgh, Usher Institute; University of Edinburgh,<br>Fagbamigbe, Adeniyi Francis; University of Aberdeen; University of Ibadan, Department of Epidemiology and Medical Statistics<br>Simpson, Colin; Victoria University of Wellington<br>Robertson, Chris; University of Strathclyde, Department of Mathematics and Statistics<br>Hippisley-Cox, Julia; University of Oxford, Nuffield Department of Primary Care Health Sciences<br>Sheikh, Aziz; University of Edinburgh, Division of Community Health Sciences

Keywords:	Health informatics < BIOTECHNOLOGY & BIOINFORMATICS, RESPIRATORY MEDICINE (see Thoracic Medicine), Epidemiology < INFECTIOUS DISEASES

SCHOLARONE™
Manuscripts

I, the Submitting Author has the right to grant and does grant on behalf of all authors of the Work (as defined in the below author licence), an exclusive licence and/or a non-exclusive licence for contributions from authors who are: i) UK Crown employees; ii) where BMJ has agreed a CC-BY licence shall apply, and/or iii) in accordance with the terms applicable for US Federal Government officers or employees acting as part of their official duties; on a worldwide, perpetual, irrevocable, royalty-free basis to BMJ Publishing Group Ltd ("BMJ") its licensees and where the relevant Journal is co-owned by BMJ to the co-owners of the Journal, to publish the Work in this journal and any other BMJ products and to exploit all rights, as set out in our licence.

The Submitting Author accepts and understands that any supply made under these terms is made by BMJ to the Submitting Author unless you are acting as an employee on behalf of your employer or a postgraduate student of an affiliated institution which is paying any applicable article publishing charge ("APC") for Open Access articles. Where the Submitting Author wishes to make the Work available on an Open Access basis (and intends to pay the relevant APC), the terms of reuse of such Open Access shall be governed by a Creative Commons licence – details of these licences and which Creative Commons licence will apply to this Work are set out in our licence referred to above.

Other than as permitted in any relevant BMJ Author's Self Archiving Policies, I confirm this Work has not been accepted for publication elsewhere, is not being considered for publication elsewhere and does not duplicate material already published. I confirm all authors consent to publication of this Work and authorise the granting of this licence.

For peer review only

External validation of the QCovid 2 and 3 risk prediction algorithms for risk of COVID-19 hospitalisation and mortality in adults: national cohort study in Scotland

Dr Steven Kerr PhD, Usher Institute, The University of Edinburgh, Edinburgh, EH8 9AG, UK

Dr Tristan Millington PhD, Usher Institute, The University of Edinburgh, Edinburgh, EH8 9AG, UK *

Prof. Igor Rudan MD, Usher Institute, The University of Edinburgh, Edinburgh, UK

Prof. Colin McCowan, School of Medicine, University of St. Andrews, St Andrews, United Kingdom

Dr Holly Tibble PhD, Usher Institute, The University of Edinburgh, Edinburgh, UK

Dr Karen Jeffrey PhD, Usher Institute, The University of Edinburgh, Edinburgh, UK

Dr Adeniyi Francis Fagbamigbe, PhD, Institute of Applied Health Sciences, University of Aberdeen, AB25 2ZD, Aberdeen, UK; Department of Epidemiology and Medical Statistics, Faculty of Public Health, College of Medicine, University of Ibadan, Ibadan, Nigeria,

Prof. Colin R Simpson PhD, School of Health, Wellington Faculty of Health, Victoria University of Wellington, Wellington, New Zealand and Usher Institute, The University of Edinburgh, Edinburgh, UK

Prof. Chris Robertson PhD, Department of Mathematics and Statistics, University of Strathclyde, Glasgow, UK and Public Health Scotland, Glasgow, UK

Professor Julia Hippisley-Cox MD, Nuffield Department of Primary Care Health Sciences, University of Oxford

Prof. Aziz Sheikh MD, Usher Institute, The University of Edinburgh, Edinburgh, UK

For peer review only

* Corresponding author. Email: Tristan.millington@gmail.com

Word Count: 2840

KEY MESSAGES

What is the key question?

Did the QCovid 2 and 3 algorithms accurately predict the risk of COVID-19 hospitalisation and death in Scotland?

What is the bottom line?

We found good performance predicting COVID-19 hospitalisations and deaths in Scotland. The Harrell's C score was consistently high across all subgroups, but the algorithm overestimated the total number of COVID-19 hospitalisations and deaths.

Why read on?

It is important to validate risk prediction tools in populations outside the data that they were trained on to inform their generalisability and use in policymaking.

For peer review only

ABSTRACT

Background: The QCovid 2 and 3 algorithms are risk prediction tools developed during the second wave of the COVID-19 pandemic that can be used to predict risk of COVID-19 hospitalisation and mortality. They were trained and internally validated on data for adults in England aged 19-100 years between 8 December 2020 and 15 June 2021. QCovid 2 and 3 expand the scope of the previous QCovid algorithm by taking account of vaccination status, with QCovid 2 predicting risk for unvaccinated individuals and QCovid 3 predicting risk for vaccinated individuals.

Methods: We used the EAVE II national COVID-19 data platform consisting of individual-level data for the population of Scotland (5.4 million residents). Primary care data were linked to reverse-transcription polymerase chain reaction (RT-PCR) virology testing, hospitalisation and mortality data. We assessed the discrimination and calibration of the QCovid 2 and 3 algorithms in predicting COVID-19 hospitalisations and deaths between 8 December 2020 and 15 June 2021.

Results: Our validation dataset comprised 465,058 individuals, aged 19-100. We found the following performance metrics (95% confidence intervals) for QCovid 2 and 3: Harrell's C 0.84 (0.82, 0.86) for hospitalisation, and 0.92 (0.90, 0.94) for death, observed-expected ratio of 0.24 for hospitalisation and 0.26 for death (i.e. both the number of hospitalisations and the number of deaths were overestimated), and a Brier score of 0.0009 (0.00084, 0.00096) for hospitalisation and 0.00036 (0.00032, 0.0004) for death.

Conclusions: We found good discrimination of the QCovid 2 and 3 algorithms in Scotland, although performance was worse in higher age groups. Both the number of hospitalisations and the number of deaths were overestimated.

Funding: National Institute for Health Research (NIHR) following a commission by the Chief Medical Officer for England. EAVE II is funded by the Medical Research Council (MC_PC_19075) with the support of BREATHE: the Health Data Research Hub for Respiratory Health (MC_PC_19004), which is funded through the UK Research and Innovation Industrial Strategy Challenge Fund and delivered through Health Data Research UK. Additional support has been provided through Public Health Scotland and the Community Health and Social Care Directorate of the Scottish Government.

For peer review only

Strengths and Limitations:

- We developed a population level dataset covering 99% of the resident population in Scotland
- We validated the QCovid 2 and 3 algorithms on this dataset according to all metrics in the original paper
- We were missing some variables which may have affected our results, notably ethnicity

Keywords: COVID-19, Clinical epidemiology

For peer review only

INTRODUCTION

In December 2019, a novel coronavirus (SARS-CoV-2) emerged in Wuhan, China. The World Health Organization (WHO) declared the outbreak a public health emergency of international concern on 30 January 2020, and then a pandemic on 11 March 2020. The index case in Scotland was identified on 1 March 2020. As of 26 March 2023, there have been over 2.1 million COVID-19 cases in Scotland, with over 17,000 deaths.(1)

Algorithms have been developed to identify people who are at risk of severe COVID-19 outcomes such as hospitalisation and death and guide public health policy.[2-7] The QCovid algorithms are risk-scoring systems that predict the probability of COVID-19 hospitalisation and death. The original QCovid algorithm was commissioned by the Chief Medical Officer (CMO) for England on behalf of the UK Government. QCovid was used by the UK Government to inform policies on shielding and vaccine prioritisation for England,(2) and has been independently and externally validated in England (3) Scotland (4) and Wales (5). In Scotland, the original QCovid algorithm was found to perform well, with close correspondence between the observed and predicted risks. The Harrell's C scores for hospitalisations and deaths in males and females were 0.809 and 0.946, respectively over the first period of testing (1 March 2020 – 30 April 2020).(4) England (3) and Wales (5) had similar results, finding very good performance.

QCovid was updated at the request of the CMO for England to take account of the evolving nature of the pandemic and vaccination status.(6) This consisted of two algorithms: QCovid 2 which predicted the probability of hospitalisation and death for unvaccinated individuals, and QCovid 3 which predicted the probability of hospitalisation and death for individuals who had received one or two doses of either the ChAdOx1 nCov-19 (Oxford AstraZeneca) or BNT162b2 (Pfizer-BioNTech) vaccines. These algorithms were developed using the QResearch database.(7)

The training cohort for QCovid2 consisted of unvaccinated individuals aged 19 – 100 years, observed between 1 September 2020 and 31 May 2021. The training cohort for QCovid3 consisted of vaccinated individuals aged 19 – 100 years old, observed between 8 December 2020 and 15 June 2021.

For peer review only - http://bmjopen.bmj.com/site/about/guidelines.xhtml

Following a request from the Scottish Government, we sought to externally validate these QCovid2 and 3 algorithms for the adult Scottish population. We used a common protocol for validating these algorithms across the four UK nations.(8) We studied the performance of QCovid 2 and 3 between 8 December 2020 and 15 June 2021, the same time period that the algorithm was trained on.

METHODS

Study design

We carried out an external validation of the QCovid 2 and 3 algorithms using the Early Pandemic Evaluation and Enhanced Surveillance of COVID-19 (EAVE II) platform, which contains electronic health records for 5.4 million (~99% of the population) people in Scotland. QCovid 3 takes into account competing risks, whereas QCovid and QCovid 2 do not. Calculating absolute risk of the outcome of interest in a Cox model with competing risks comes with a significant increase in computational demand. Due to limitations on computational resources, we performed this validation using a random 10% sample of the cohort.

Datasets

We had data from all 940 Scottish primary care practices. These were linked to the Electronic Communication of Surveillance in Scotland (ECOSS; national database for all virology testing including NHS and UK Government test centre data), the Scottish Morbidity Record (record of hospitalisation data), and National Records Scotland (death certification) data as part of the EAVE II platform. A more detailed description of the data can be found in our cohort profile.[10] Table S1 shows a description of the cohort as well as the 10% sample.

Selection criteria

Any individual in the linked dataset aged between 19 and 100 years on 8 December 2020 was eligible for inclusion. Anyone who had a COVID-19 related hospitalisation before the start of follow-up for each dose was excluded from the hospitalisation analysis.

Exposures

For peer review only

Predictor variables were those used in the QCovid 2 and 3 algorithms.(6) These are detailed in Box S1. All predictor variables were taken as the most recent recorded value in the data at cohort entry.

Outcomes

The primary outcomes were time to COVID-19 hospitalisation, and time to COVID-19 death. COVID-19 hospitalisation was defined as hospitalisation with reverse transcription polymerase chain reaction (RT-PCR) positive COVID-19 test within 28 days prior to or admission with ICD-10 (International Classification of Disease version 10) codes for COVID-19 (U07.1, U07.2). COVID-19 death was defined as all-cause death within 28 days of a post-positive RT-PCR test, or death with ICD-10 codes for COVID-19 on the death certificate from National Records Scotland.

Missing data

We could not obtain data relating to some conditions used in the QCovid 2 and 3 algorithms. We did not have reliable data available indicating whether the individual had a bone marrow or stem cell transplant in the last six months, whether they had received radiotherapy in the last six months, whether they had been prescribed immunosuppressants, oral steroids or anti-leukotriene or long acting beta2-agonists four or more times in the last six months, whether they had irritable bowel syndrome, and whether they had received a solid organ transplant. The values of these variables were set to "none". We had data on the type of diabetes, but not if it was controlled. If the individual had type 1 diabetes, we took them to be in the uncontrolled category, and if they had type 2 diabetes, we took them to be in the controlled category, as these were the most populous categories in the training data.(6) For all other comorbidities/treatments, a missing value was taken to indicate absence of that comorbidity/treatment.

Reliable ethnicity data were not available, and all individuals were assigned to "White British". In the 2011 Scottish census, 96% of the population was in this ethnic group.(9) The most fine-grained residential location information available in our dataset was data zone, which is a geographical designation comprising of groups of UK Census output areas. Output areas typically consist of ~300 people, whereas data zones typically consist of 500–1,000 people.[11] Townsend Deprivation Scores [12] for each output area were obtained from the 2011 UK census.(10) We took the median value of Townsend Deprivation Scores for the

For peer review only

output areas comprising each data zone to get a deprivation score for each data zone. Missing values for Townsend Deprivation Scores were replaced with the mean value for the cohort. Missing values in the housing category variable were taken to indicate the individual was neither homeless, nor resident in a care home.

We used single imputation by chained equations (11) to impute missing values for body mass index (BMI). There is some evidence of an association between lower levels of socioeconomic status and higher BMI in economically developed countries.(12) Sex is known to be associated with BMI, as is coronary artery disease and diabetes.(13)

There were no missing values for any of the other independent variables.

Model validation

To calculate the risk for those who were unvaccinated, the baseline survival rate was provided on the following days: 0, 30, 60, 90, 183, 210, 240. We linearly interpolated the logarithm of the baseline survival rate to obtain values for intermediate days. We calculated probability of event for an individual either on the day of event, or the day of censoring.

We applied versions 2 and 3 of the QCovid algorithm to a random 10% sample of our cohort, and computed Harrell's Concordance,(14) the Brier score, Royston's D,(15) R^2 (16) and observed-expected (OE) ratio for the period 8 December 2020 to 15 June 2021. As well as calculating these metrics for the entire cohort sample, we also calculated them for several sub-cohorts. These sub-cohorts consisted of unvaccinated individuals, individuals followed up after first and second dose, and the age ranges, 19-64, 65-79 and 80-100.

Harrell's Concordance is a performance metric that characterises the tendency for people with higher hazard rates to have earlier events. It takes values between 0 and 1, with 0 indicating poorer performance and 1 indicating better performance. The Brier score is a measure of forecast accuracy that is equal to the mean squared prediction error in the case of a binary outcome variable. It takes values greater than 0, with zero indicating the best performance. Royston's D is a measure of 'separation' between survival curves. Higher values of Royston's D indicate predicted hazard rates have more discriminative ability. R^2 is a measure of the proportion of variation in survival time explained by the model. It takes values between 0 and 1, with 0 indicating poorer performance and 1 indicating better

For peer review only

performance. We calculated R^2 for survival models as defined in (16). The OE ratio is the number of observed events divided by the expected number of events predicted by the model. A value of 1 indicates that the total number of events is exactly correctly predicted, and a value less than 1 indicates that the total number of events predicted was greater than observed. We made plots of observed vs expected risk on the day of event/censoring by vigintiles (20 groups) of predicted hazard rate. We also calculated recalibrated Brier scores and made re-calibrated plots of observed vs expected risk by scaling predicted risks by a multiplicative constant so that expected total number of events predicted was equal to observed total number of events.

Reporting

This study is reported in accordance with the Transparent reporting of a multivariable prediction model for individual prognosis or diagnosis (TRIPOD) guidelines. (17)

Patient and public involvement

The EAVE II Public Advisory Group reviewed the online QCovid tools and the preliminary results from this paper. Although overall in favour of this work, they expressed some concern at the lack of ethnicity data for our results, and that the online tools would need significant editing to be accessible to the publics.

Ethics and permissions

Ethical permission for this study was granted by the South East Scotland Research Ethics Committee 02 [12/SS/0201]. The Public Benefit and Privacy Panel Committee of Public Health Scotland, approved the linkage and analysis of the de–identified datasets for this project [1920-0279].

RESULTS

In our 10% cohort sample, there were 763 COVID-19 hospitalisations in the validation period, and 393 COVID-19 deaths. Table 1 shows the OE ratio, Harrell's C, Brier score, Royston's D, R^2 and recalibrated Brier score with outcomes of COVID-19 hospitalisation and COVID-19 death for the entire cohort sample, as well as subgroups stratified by follow up after different vaccine doses. Table 2 shows the same statistics, stratified by age group. Figure 1 plots the observed and expected risk of hospitalisation and death by vigintiles of the hazard rate (HR) for the entire sample, the subset who were unvaccinated and those during

For peer review only

follow up after first and second dose, and Figure 2 shows the same set of figures but recalibrated with the actual number of events. The vigintiles were numbered so that higher vigintiles had a higher hazard rate (i.e., those in vigintile 1 were in the lowest hazard rate category, those in vigintile 20 were in the higher hazard rate category).

Vaccine dose

Focusing firstly on hospitalisations stratified by vaccine dose (shown in the top half of Table 1), according to Harrell's C, Royston's D and the R^2 metric, performance was similar across all sub-groups, but better for the entire sample cohort. This is because combining data unvaccinated with vaccinated people created a large number of additional concordant pairs. Both the Brier score and the recalibrated Brier scores were noticeably worse for the unvaccinated than for those who had one or two vaccine doses, or the entire sample cohort.

In terms of COVID-19 deaths (shown in the bottom half of Table 1), we found that the model performed similarly according to Harrell's C across the vaccine dose follow-up categories. Again, across the other metrics bar the OE ratio, performance was worse for the unvaccinated than for those who had one or two vaccine doses, or for the entire sample cohort.

For both events, the performance according to Harrell's C was very good, and was similar to that in the original study on their validation cohort (0.84 (0.82, 0.86) for hospitalisation in this paper vs 0.85 in the original study, and 0.92 (0.90 – 0.94) in this paper vs 0.93 in the original study) (6). The D statistic performance in this paper was worse than in the original study (2.21 (2.022, 2.218) vs 2.83 (2.59, 3.08) for hospitalisation and 2.77 (2.61, 2.93) vs 3.46 (3.19, 3.73) for death).

Age

Looking at the results for COVID-19 hospitalisation stratified by age (shown in top half of Table 2), the model performed less well for the older age groups (65 – 79, ≥80) compared to the younger group (18 – 64) according to all metrics, except for the OE ratio. This pattern is also repeated for COVID-19 death stratified by age (shown in the bottom half of Table 2).

Vigintile plots

For peer review only

Figure 1 shows a set of vigintile plots comparing the predicting risk to the observed risk for the full cohort and two sub cohorts – those who were unvaccinated and those with a first and second dose. Figure 2 shows the same set of vigintile plots, but with the risks recalibrated.

The vigintile plots on the left-hand side of Figure 1 show the model's performance in terms of predicting COVID-19 hospitalisations (left hand side) and deaths (right hand size). For both events, the models tended to overestimate the events across all stratifications. The estimates tended to be worse for the higher risk vigintiles.

Figure 2 shows the same plots after we recalibrated the models. After this we found that most of the vigintiles had a good estimation of the number of events after recalibration. Across the stratifications, there was no consistent pattern of over-estimating or underestimating the number of events for specific vigintiles. The predictions for the ≥80 years sub-cohort, particularly for death, were not as good as for the other cohorts. This may have been due to the small sample size combined with the larger risk scores.

DISCUSSION

Statement of principal findings

In this external validation of the QCovid 2 and 3 algorithms in Scotland, we found the algorithms performed well with regards to discrimination as measured by the C statistic. The total number of events was overestimated for hospitalisations and deaths across all subgroups. Performance was broadly similar to that in the original paper.(6)

Strengths and Limitations

Our study had a number of important strengths. We developed a unique linked dataset covering 99% of the population resident in Scotland. The EAVE II database (18) is one of the few national individual patient-level linked research databases in the world.(19) We evaluated the performance of the QCovid 2 and 3 algorithms according to all metrics used in the original paper (6) and in the common protocol agreed between the four UK nations.(8)

However, our work has several limitations. Firstly, we did not have access to ethnicity data, so all individuals were set to 'White British' ethnicity. We believe modal substitution for ethnicity was reasonable because the most recent Scottish census indicated that 96% of the residents of Scotland identified their ethnicity as 'White'.(9) There was significant

For peer review only

missingness in the BMI data, with 2,495,504 (55.6%) missing values. We used single imputation by chained equations to impute these missing values for BMI. Multiple imputation was not used due to limitations on computational resources. The most fine-grained residential location information available in our dataset was data zone, which typically consists of multiple 2011 UK census output areas. We took the median value of the Townsend Deprivation Scores for the output areas comprising each data zone to get a deprivation score for each data zone. Missing values of Townsend Deprivation Scores were replaced with the average value for the cohort. Higher levels of deprivation as measured by Townsend Deprivation Scores was associated with increased predicted risk of COVID-19 hospitalisation and death in the QCovid 2 and 3 algorithms. We also did not have data for several clinical risk variables, so individuals were assigned to the category for absence of the condition. This will have had the effect of under-estimating risk in people with these characteristics. We also validated on a 10% sample randomly chosen rather than the full EAVE II cohort due to limitations on computational resources. This led to there being few samples in some of the higher risk groups, and wider confidence intervals on the metrics.

Interpretation

The QCovid 2 and 3 risk prediction algorithms performed well in the Scottish population in the period they were trained for.

Implications for policy, practise and research

Our results indicate that QCovid 2 and 3 would have been appropriate for use as a risk prediction tool for COVID-19 hospitalisation and death in Scotland during our study period.

CONCLUSION

Risk prediction tools are valuable for identifying individuals at the highest risk of experiencing severe outcomes, and can be used by policy makers to help mitigate these risks. However, these tools must be externally validated on cohorts that were not used to train them. We evaluated the QCovid2 and 3 algorithms in Scotland using a variety of metrics and across several sub-groups. We found good performance overall, and many measures were similar to those in the original paper.

Acknowledgements: The authors would like to thank staff at Public Health Scotland,

For peer review only

Albasoft Ltd, the general practices that contributed data to the study and the EAVE II Collaborators. AS, JM and CR serve on The Scottish Government's COVID-19 Chief Medical Officer's Advisory Group and the New and Emerging Respiratory Virus Threats Advisory Group (NERVTAG) Risk Stratification Subgroup.

Data sharing: All code, metadata and documentation for this project is publicly available at https://github.com/EAVE-II/QCovid2-3-validation. A data dictionary is available at https://github.com/EAVE-II/EAVE-II-data-dictionary. Most of the data that were used in this study are highly sensitive and will not be made available publicly.

Authors and contributors: AS and JHC conceptualised the study. SK carried out the formal analysis. TM wrote an initial draft of the manuscript. All authors assisted with review and editing. CR and TM have verified the underlying data.

Declaration of interest: Dr. Hippisley-Cox reports grants from MRC, grants from Wellcome Trust, grants from NIHR, during the conduct of the study; JHC is a founder and shareholder of ClinRisk ltd and was its medical director until 31st May 2019. ClinRisk Ltd produces open and closed source software to implement clinical risk algorithms (outside this work) into clinical computer systems. JHC was chair of the NERVTAG risk stratification subgroup and a member of SAGE COVID-19 groups and the NHS group advising on prioritisation of use of monoclonal antibodies in COVID-19 infection. Dr. Sheikh reports grants from NIHR, grants from MRC, grants from HDR UK, during the conduct of the study. All other authors report no conflict of interest.

Table 1: Performance metrics (95% confidence intervals) stratified by vaccination status for COVID-19 hospitalisation and death.

Group	Observed-expected ratio	C	Brier Score	Royston's D	Recalibrated Brier Score	R^2
**Hospitalisation**						
Everyone	0.24	0.84 (0.82 - 0.86)	0.0009 (0.00084 - 0.00096)	2.12 (2.022 - 2.218)	0.00091 (0.00085 - 0.00097)	0.52 (0.49 - 0.54)
Unvaccinated	0.23	0.73 (0.71 - 0.75)	0.00209 (0.00193 - 0.00225)	1.33 (1.2124 - 1.4476)	0.00211 (0.00195 - 0.00227)	0.3 (0.26 - 0.33)
First and second dose vaccinated	0.33	0.72 (0.66 - 0.78)	0.00015 (0.00013 - 0.00017)	1.43 (1.136 - 1.724)	0.00015 (0.00013 - 0.00017)	0.33 (0.23 - 0.42)
**Death**						
Total population	0.26	0.92 (0.9 - 0.94)	0.00036 (0.00032 – 0.004)	2.77 (2.6132 - 2.9268)	0.00036 (0.00032 – 0.0004)	0.65 (0.62 - 0.67)
Unvaccinated	0.34	0.88 (0.86 - 0.9)	0.00077 (0.00069 - 0.00085)	2.35 (2.1736 - 2.5264)	0.00077 (0.00069 - 0.00085)	0.57 (0.53 - 0.6)

For peer review only

First and second dose vaccinated	0.13	0.94 (0.92 - 0.96)	0.00001 (0.00008 - 0.00012)	3.09 (2.7372 - 3.4428)	1.0001 (0.00008 - 0.00012)	0.7 (0.64 - 0.74)

Table 2: Performance metrics (95% confidence intervals) for COVID-19 hospitalisation and death stratified by age group.

Age Group	Observed -expected ratio	C	Brier Score	Royston's D	Recalibrated Brier Score	R^2
**Hospitalisation**						
Age 18-64	0.21	0.82 (0.8 - 0.84)	0.00067 (0.00061 - 0.00073)	1.9 (1.7628 - 2.0372)	0.00068 (0.00062 - 0.00074)	0.46 (0.42 - 0.5)
Age 65-79	0.29	0.80 (0.78 - 0.82)	0.00112 (0.00098 - 0.00126)	1.70 (1.504 - 1.896)	0.00113 (0.00099 - 0.00127)	0.41 (0.35 - 0.46)
Age 80+	0.27	0.78 (0.76 - 0.8)	0.00255 (0.0022 - 0.0029)	1.55 (1.3344 - 1.7656)	0.00258 (0.00223 - 0.00293)	0.36 (0.3 - 0.43)
**Death**						
Age 18-64	0.15	0.85 (0.81 - 0.89)	0.00007 (0.00005 – 0.00009)	1.96 (1.5288 - 2.3912)	0.00007 (0.00005 – 0.00009)	0.48 (0.36 - 0.58)
Age 65-79	0.28	0.82 (0.8 - 0.84)	0.00052 (0.00042 - 0.00062)	1.77 (1.476 - 2.064)	0.00052 (0.00042 - 0.00062)	0.43 (0.34 - 0.51)
Age 80+	0.3	0.73 (0.71	0.00309 (0.0027 - 0.00348)	1.23 (1.034 - 1.426)	0.00311 (0.0027 - 0.00352)	0.26 (0.2 - 0.33)

For peer review only

		- 0.75)				

Figure 1: Vigintile plots of expected vs observed risks for the cohort and a selection of sub-cohorts.

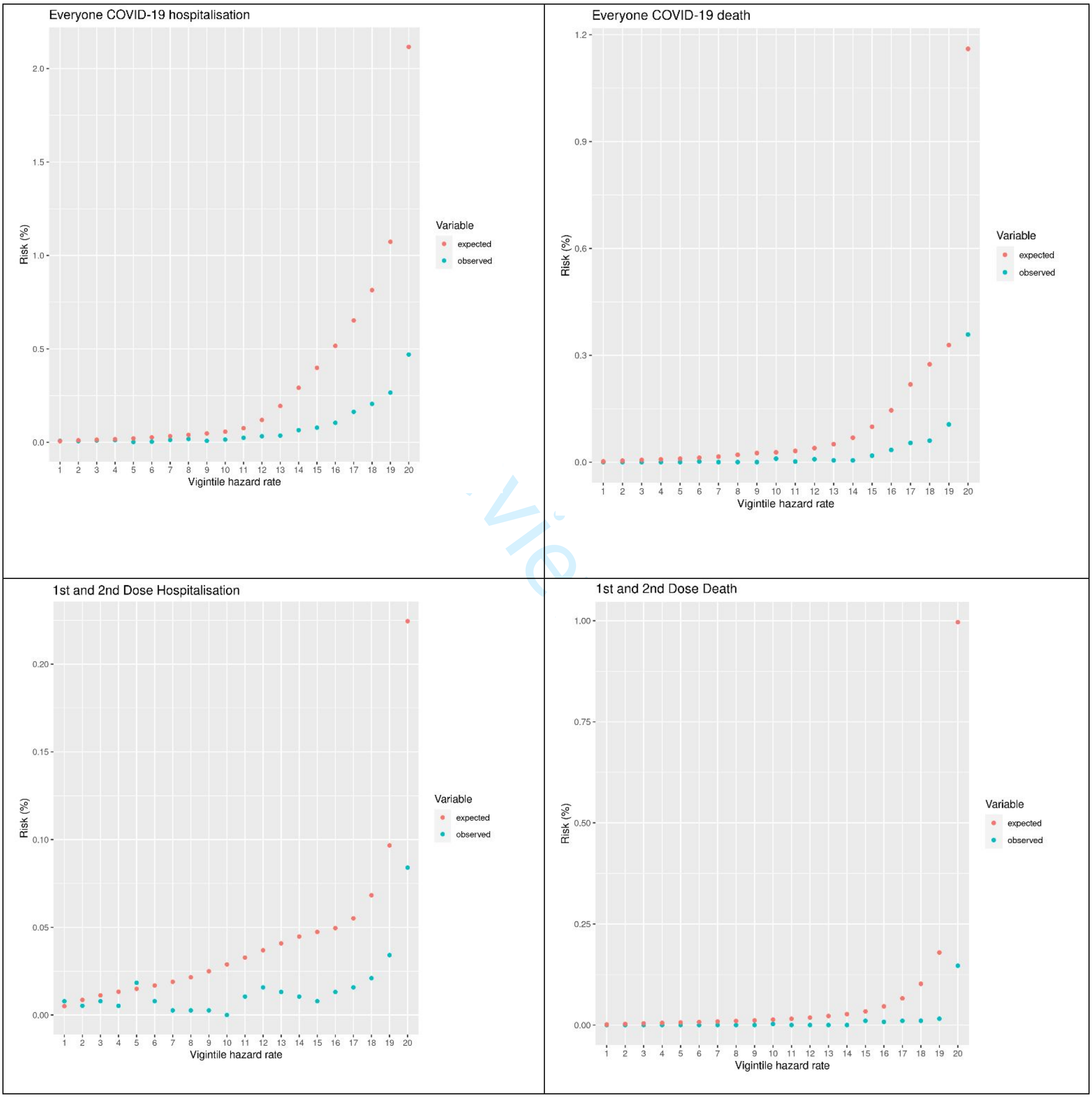

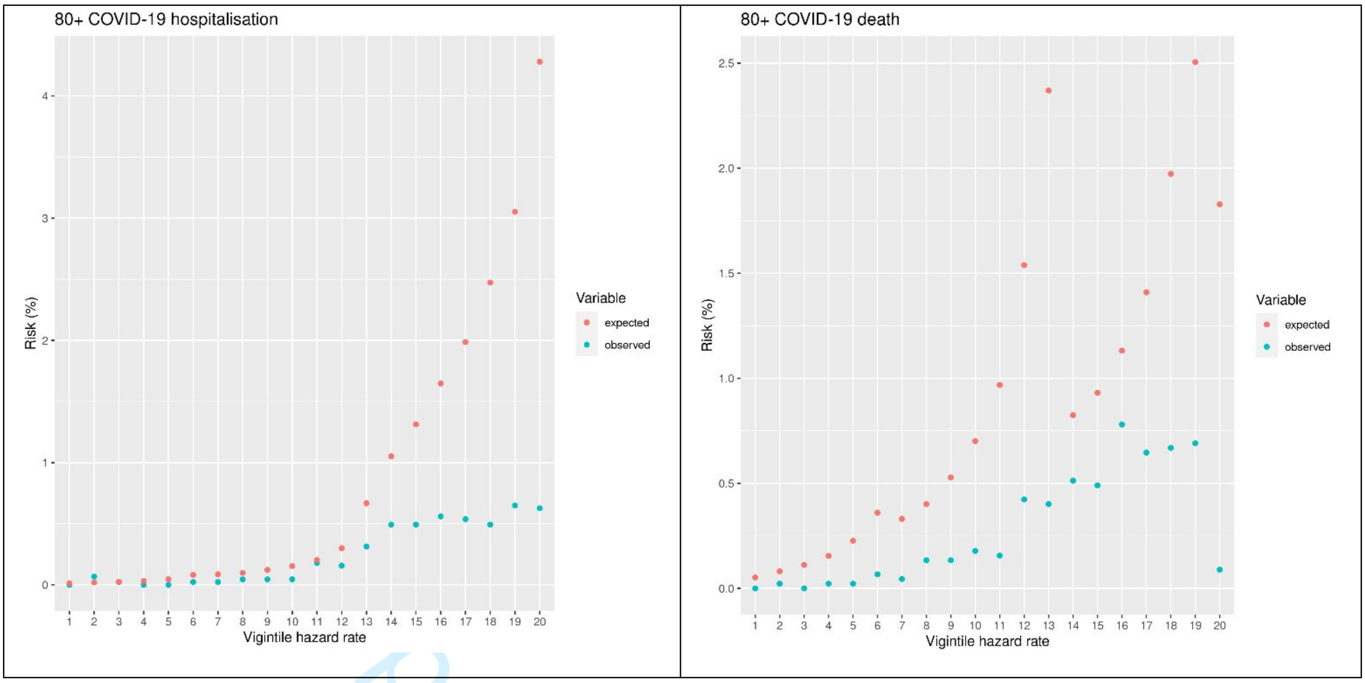

Figure 2: Vigintile plots of expected vs observed recalibrated risks for the cohort and a selection of sub-cohorts.

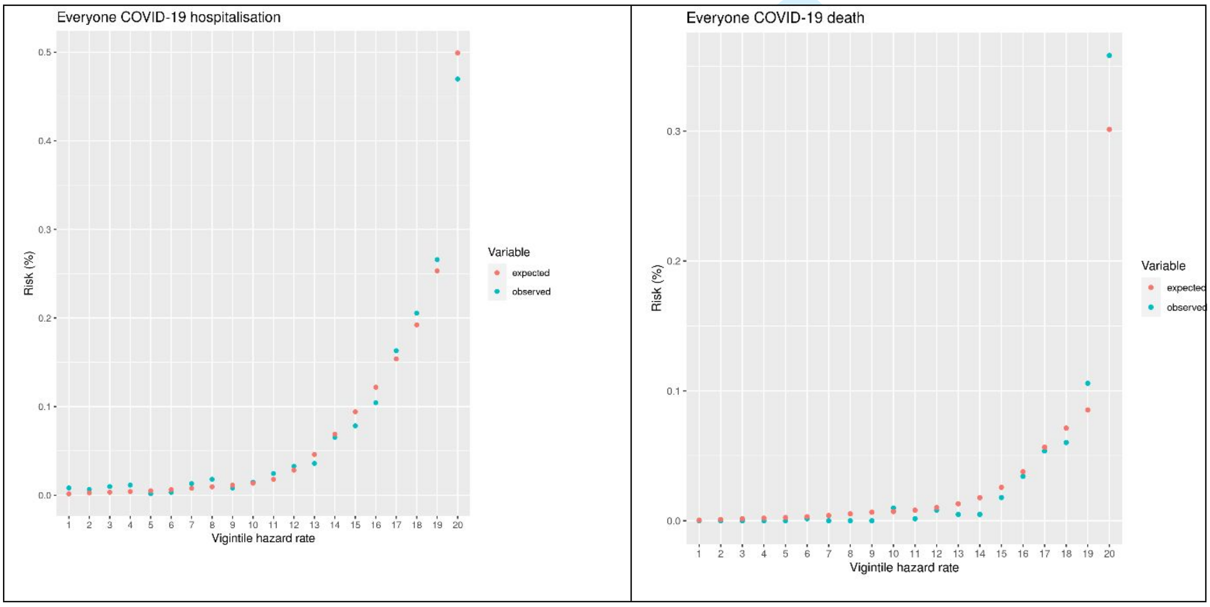

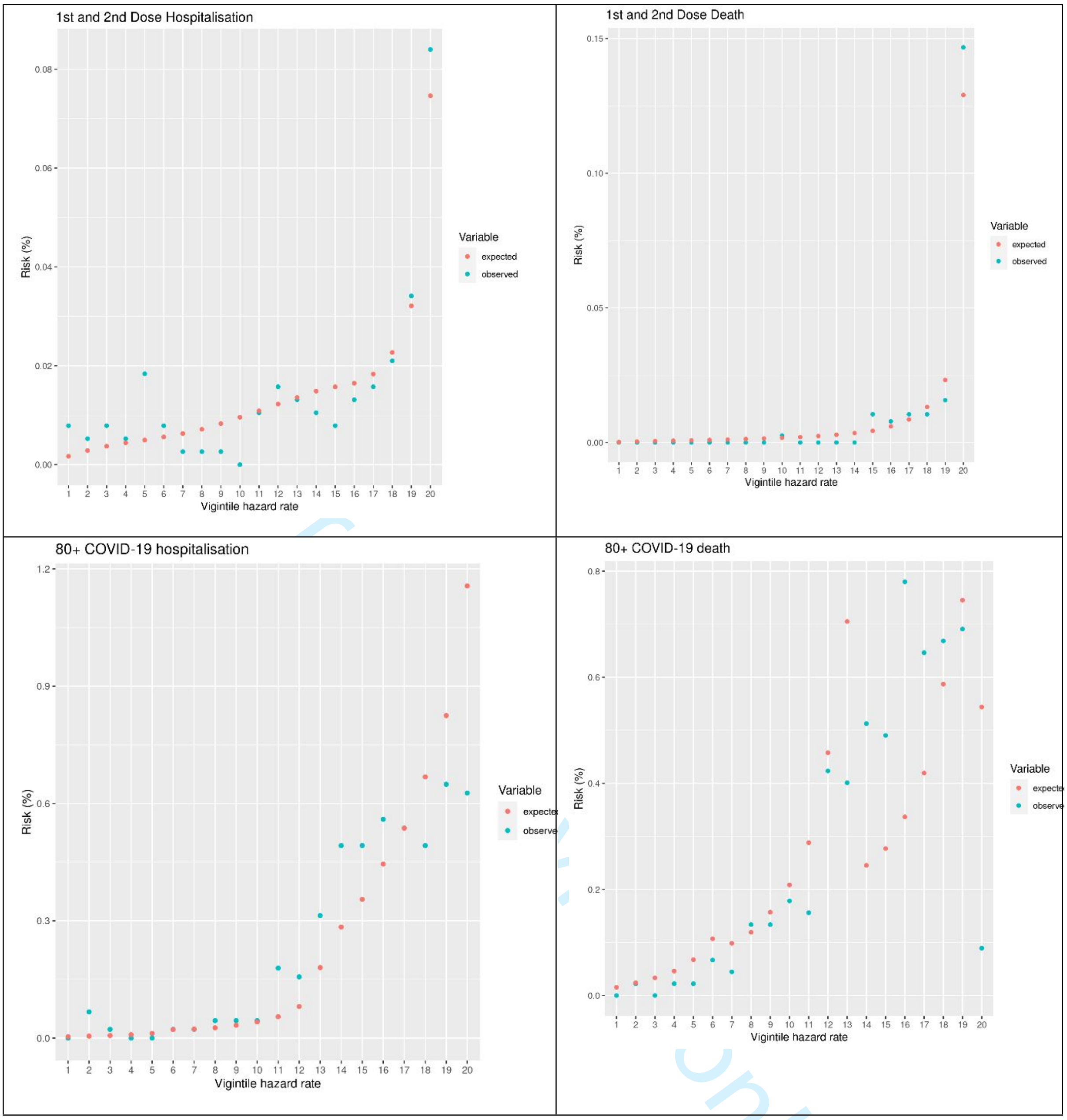

References

1. Deaths involving coronavirus (COVID-19) in Scotland 2023 [Available from: https://www.nrscotland.gov.uk/statistics-and-data/statistics/statistics-by-theme/vital-events/general-publications/weekly-and-monthly-data-on-births-and-deaths/deaths-involving-coronavirus-covid-19-in-scotland

2. New technology to help identify those at high risk from COVID-19 [Available from: https://www.gov.uk/government/news/new-technology-to-help-identify-those-at-high-risk-from-covid-19.

3. Nafilyan V, Humberstone B, Mehta N, Diamond I, Coupland C, Lorenzi L, et al. An external validation of the QCovid risk prediction algorithm for risk of mortality from COVID-19 in adults: a national validation cohort study in England. The Lancet Digital Health. 2021;3(7):e425-e33.

4. Simpson CR, Robertson C, Kerr S, Shi T, Vasileiou E, Moore E, et al. External validation of the QCovid risk prediction algorithm for risk of COVID-19 hospitalisation and mortality in adults: national validation cohort study in Scotland. Thorax. 2022;77(5):497-504.
5. Lyons J, Nafilyan V, Akbari A, Davies G, Griffiths R, Harrison EM, et al. Validating the QCOVID risk prediction algorithm for risk of mortality from COVID-19 in the adult population in Wales, UK. Int J Popul Data Sci. 2020;5(4):1697.
6. Hippisley-Cox J, Coupland CA, Mehta N, Keogh RH, Diaz-Ordaz K, Khunti K, et al. Risk prediction of covid-19 related death and hospital admission in adults after covid-19 vaccination: national prospective cohort study. BMJ. 2021;374:n2244.
7. Hippisley-Cox J, Stables D, Pringle M. QRESEARCH: a new general practice database for research. Inform Prim Care. 2004;12(1):49-50.
8. Kerr S, Robertson C, Nafilyan V, Lyons RA, Kee F, Cardwell CR, et al. Common protocol for validation of the QCOVID algorithm across the four UK nations. BMJ Open. 2022;12(6):e050994.
9. Scotland's Census - Ethnicity 2021 [Available from: https://www.scotlandscensus.gov.uk/census-results/at-a-glance/ethnicity/.
10. 2011 UK Census Townsend Deprivation Scores [Available from: https://statistics.ukdataservice.ac.uk/dataset/2011-uk-townsend-deprivation-scores.
11. Little RJ, Rubin DB. Single imputation methods. Statistical analysis with missing data. 2002:59-74.
12. McLaren L. Socioeconomic status and obesity. Epidemiologic reviews. 2007;29(1):29-48.
13. Bays HE, Chapman RH, Grandy S. The relationship of body mass index to diabetes mellitus, hypertension and dyslipidaemia: comparison of data from two national surveys. Int J Clin Pract. 2007;61(5):737-47.
14. Harrell FE, Jr., Califf RM, Pryor DB, Lee KL, Rosati RA. Evaluating the yield of medical tests. Jama. 1982;247(18):2543-6.
15. Royston P, Sauerbrei W. A new measure of prognostic separation in survival data. Stat Med. 2004;23(5):723-48.
16. Royston P. Explained variation for survival models. The Stata Journal. 2006;6(1):83-96.
17. Moons KG, Altman DG, Reitsma JB, Ioannidis JP, Macaskill P, Steyerberg EW, et al. Transparent Reporting of a multivariable prediction model for Individual Prognosis or Diagnosis (TRIPOD): explanation and elaboration. Ann Intern Med. 2015;162(1):W1-73.
18. Simpson CR, Robertson C, Vasileiou E, McMenamin J, Gunson R, Ritchie LD, et al. Early Pandemic Evaluation and Enhanced Surveillance of COVID-19 (EAVE II): protocol for an observational study using linked Scottish national data. BMJ Open. 2020;10(6):e039097.
19. Simpson CR, Beever D, Challen K, De Angelis D, Fragaszy E, Goodacre S, et al. The UK's pandemic influenza research portfolio: a model for future research on emerging infections. Lancet Infect Dis. 2019;19(8):e295-e300.

For peer review only

Table S1: Summary statistics for the full cohort and 10% sample

Characteristics	Levels	Entire cohort	10% cohort sample
Total		4650585 (100.0)	465058 (100.0)
COVID-19 Deaths		4049 (0.1)	393 (0.1)
COVID-19 Hospitalisations		9547 (0.2)	763 (0.2)
Sex	Female	2358990 (50.7)	235821 (50.7)
	Male	2291595 (49.3)	229237 (49.3)
Age	Mean (SD)	50.3 (18.8)	50.3 (18.9)
Age group	19-39	1581219 (34.0)	158052 (34.0)
	40-59	1533605 (33.0)	152985 (32.9)
	60-74	978827 (21.0)	98190 (21.1)
	75-100	556934 (12.0)	55831 (12.0)
SIMD quintile	1 - Most deprived	925925 (19.9)	92546 (19.9)
	2	918884 (19.8)	92073 (19.8)
	3	918091 (19.7)	91868 (19.8)
	4	921838 (19.8)	92219 (19.8)
	5 - Least deprived	924963 (19.9)	92392 (19.9)
	(Missing)	40884 (0.9)	3960 (0.9)
Urban rural classification	Large Urban Areas	1671719 (35.9)	166887 (35.9)
	Other Urban Areas	1652152 (35.5)	165274 (35.5)
	Accessible Small Towns	424031 (9.1)	42739 (9.2)
	Remote Small Towns	221513 (4.8)	22271 (4.8)
	Accessible Rural	413436 (8.9)	41170 (8.9)
	Remote Rural	226850 (4.9)	22757 (4.9)
	(Missing)	40884 (0.9)	3960 (0.9)
Ethnicity	Asian	102490 (2.2)	10250 (2.2)
	Black	25123 (0.5)	2567 (0.6)
	Mixed	21139 (0.5)	2148 (0.5)

For peer review only

	Other	23584 (0.5)	2383 (0.5)
	Unknown	1562888 (33.6)	156408 (33.6)
	White	2915361 (62.7)	291302 (62.6)
Number of risk groups	0	2725853 (58.6)	272930 (58.7)
	1	1249848 (26.9)	125016 (26.9)
	2	434728 (9.3)	43153 (9.3)
	3 - 4	210691 (4.5)	21024 (4.5)
	5+	29465 (0.6)	2935 (0.6)
BMI	15-18.5	48651 (1.0)	4772 (1.0)
	18.5-25	543001 (11.7)	54377 (11.7)
	25-30	674760 (14.5)	67921 (14.6)
	30-35	431788 (9.3)	43170 (9.3)
	35-40	198273 (4.3)	19835 (4.3)
	40+	133917 (2.9)	13226 (2.8)
	(Missing)	2620195 (56.3)	261757 (56.3)
Housing category	Neither	4624584 (99.4)	462533 (99.5)
	Care home	25217 (0.5)	2457 (0.5)
	Homeless	784 (0.0)	68 (0.0)
Learning disability/Down's syndrome	Neither	4600228 (98.9)	460016 (98.9)
	Down's syndrome	1526 (0.0)	144 (0.0)
	Learning disability	48831 (1.0)	4898 (1.1)
Chronic Kidney Disease	No CKD	4484131 (96.4)	448600 (96.5)
	CKD 3	166431 (3.6)	16455 (3.5)
	CKD 4	19 (0.0)	*
	CKD 5	4 (0.0)	*
Atrial Fibrillation		114239 (2.5)	11502 (2.5)
Asthma		494549 (10.6)	49499 (10.6)
Blood cancer		20162 (0.4)	2049 (0.4)

For peer review only

Congestive Cardiac Failure		46961 (1.0)	4748 (1.0)
Cerebral Palsy		5521 (0.1)	529 (0.1)
Coronary heart disease		191035 (4.1)	19132 (4.1)
Liver cirrhosis		10882 (0.2)	1116 (0.2)
Congenital heart disease		18969 (0.4)	1854 (0.4)
COPD		118884 (2.6)	11922 (2.6)
Dementia		23258 (0.5)	2202 (0.5)
Diabetes Type 1		65238 (1.4)	6434 (1.4)
Diabetes Type 2		205608 (4.4)	20446 (4.4)
Epilepsy		4032 (0.1)	386 (0.1)
Hip, wrist, spine, humerus fracture		1638 (0.0)	140 (0.0)
HIV/AIDS		18937 (0.4)	1865 (0.4)
Severe combined immunodeficiency syndrome		9707 (0.2)	987 (0.2)
Neurological conditions		6916 (0.1)	668 (0.1)
Parkinsons disease		23496 (0.5)	2367 (0.5)
Pulmonary hypertension		11937 (0.3)	1212 (0.3)
Cystic fibrosis, bronchiectasis or alveolitis		88090 (1.9)	8852 (1.9)
Peripheral vascular disease		5452 (0.1)	538 (0.1)
SLE or rheumatoid arthritis		19750 (0.4)	1892 (0.4)
Lung, oral cancer		205 (0.0)	8 (0.0)

For peer review only

Severe mental illness		115619 (2.5)	11431 (2.5)
Sickle cell disease or combined immune deficiency syndrome		41948 (0.9)	4198 (0.9)
Stroke, transient ischaemic attack		22733 (0.5)	2309 (0.5)
Venous thromboembolism		265285 (5.7)	26697 (5.7)

For peer review only

Box S1: Predictor variables in the QCovid 3 algorithm

- Vaccine dose
- Age in years (continuous)
- Sex
- Ethnicity in ten categories (Bangladeshi, Black African, Black Caribbean, Chinese, Indian, Mixed, Pakistani, White British, White other, Other, unknown)
- Townsend deprivation score (continuous)
- Body Mass Index (kg/m2)
- Accommodation (Neither homeless nor care home, care home or nursing home)
- Chronic kidney disease (CKD) – (no CKD, CKD3, CKD4, CKD5, unknown)
- Learning disability (No learning disability, Down's Syndrome, other learning disability)
- Chemotherapy in last 12 months (none, Chemotherapy group A, B, C, unknown)
- Blood Cancer
- Bone Marrow transplant in the past 6 months
- Rare Lung Diseases (cystic fibrosis, bronchiectasis, or alveolitis)
- Respiratory cancer
- Radiotherapy in last 6 months
- Solid organ transplant
- Prescribed immunosuppressant medication by GP
- Prescribed leukotriene or long-acting beta blockers
- Prescribed regular prednisolone
- Sickle cell disease
- Diabetes
- Chronic obstructive pulmonary disease (COPD)
- Asthma
- Rare pulmonary diseases
- Pulmonary hypertension or pulmonary fibrosis
- Coronary heart disease
- Stroke
- Atrial Fibrillation
- Congestive cardiac failure
- Venous thromboembolism
- Peripheral vascular disease
- Congenital heart disease
- Dementia
- Heart Failure
- Parkinson's disease
- Epilepsy
- Rare neurological conditions
- Cerebral palsy
- Severe mental illness (bipolar disorder, schizophrenia, severe depression)
- Osteoporotic fracture
- Rheumatoid arthritis or Systemic lupus erythematosus
- Cirrhosis of the liver
- Inflammatory Bowel Disease
- HIV/AIDS
- Severe Combined Immunodeficiency
- Previous Positive SARS-CoV-2 positive test result
- Background rate of RT-PCR positivity per 100,000 people

For peer review only

Reporting checklist for prediction model development/validation.

Based on the TRIPOD guidelines.

Instructions to authors

Complete this checklist by entering the page numbers from your manuscript where readers will find each of the items listed below.

Your article may not currently address all the items on the checklist. Please modify your text to include the missing information. If you are certain that an item does not apply, please write "n/a" and provide a short explanation.

Upload your completed checklist as an extra file when you submit to a journal.

In your methods section, say that you used the TRIPOD reporting guidelines, and cite them as:

Collins GS, Reitsma JB, Altman DG, Moons KG. Transparent reporting of a multivariable prediction model for individual prognosis or diagnosis (TRIPOD): The TRIPOD statement.

		Reporting Item	Page Number
**Title**			
	#1	Identify the study as developing and / or validating a multivariable prediction model, the target population, and the outcome to be predicted.	1
**Abstract**			

For peer review only

	#2	Provide a summary of objectives, study design, setting, participants, sample size, predictors, outcome, statistical analysis, results, and conclusions.	3
**Introduction**			
	#3a	Explain the medical context (including whether diagnostic or prognostic) and rationale for developing or validating the multivariable prediction model, including references to existing models.	5
	#3b	Specify the objectives, including whether the study describes the development or validation of the model or both.	6
**Methods**			
Source of data	#4a	Describe the study design or source of data (e.g., randomized trial, cohort, or registry data), separately for the development and validation data sets, if applicable.	6
Source of data	#4b	Specify the key study dates, including start of accrual; end of accrual; and, if applicable, end of follow-up.	6, 8
Participants	#5a	Specify key elements of the study setting (e.g., primary care, secondary care, general	6

For peer review only

		population) including number and location of centres.	
Participants	#5b	Describe eligibility criteria for participants.	6
Participants	#5c	Give details of treatments received, if relevant	NA
Outcome	#6a	Clearly define the outcome that is predicted by the prediction model, including how and when assessed.	7
Outcome	#6b	Report any actions to blind assessment of the outcome to be predicted.	No actions necessary
Predictors	#7a	Clearly define all predictors used in developing or validating the multivariable prediction model, including how and when they were measured	Supplementary material 4
Predictors	#7b	Report any actions to blind assessment of predictors for the outcome and other predictors.	No actions necessary
Sample size	#8	Explain how the study size was arrived at.	6
Missing data	#9	Describe how missing data were handled (e.g., complete-case analysis, single imputation, multiple imputation) with details of any imputation method.	7

Statistical analysis methods	#10a	If you are developing a prediction model describe how predictors were handled in the analyses.	NA – not developing a model
Statistical analysis methods	#10b	If you are developing a prediction model, specify type of model, all model-building procedures (including any predictor selection), and method for internal validation.	NA – not developing a model
Statistical analysis methods	#10c	If you are validating a prediction model, describe how the predictions were calculated.	8
Statistical analysis methods	#10d	Specify all measures used to assess model performance and, if relevant, to compare multiple models.	8
Statistical analysis methods	#10e	If you are validating a prediction model, describe any model updating (e.g., recalibration) arising from the validation, if done	9
Risk groups	#11	Provide details on how risk groups were created, if done.	6
Development vs. validation	#12	For validation, identify any differences from the development data in setting, eligibility criteria, outcome, and predictors.	2

For peer review only

Results

Participants	#13a	Describe the flow of participants through the study, including the number of participants with and without the outcome and, if applicable, a summary of the follow-up time. A diagram may be helpful.	
Participants	#13b	Describe the characteristics of the participants (basic demographics, clinical features, available predictors), including the number of participants with missing data for predictors and outcome.	Supplementary material p1
Participants	#13c	For validation, show a comparison with the development data of the distribution of important variables (demographics, predictors and outcome).	NA – we are validating an external algorithm trained on external data we do not have access to
Model development	#14a	If developing a model, specify the number of participants and outcome events in each analysis.	NA – not developing a model
Model development	#14b	If developing a model, report the unadjusted association, if calculated between each candidate predictor and outcome.	NA – not developing a model
Model specification	#15a	If developing a model, present the full prediction model to allow predictions for	NA – not developing a model

For peer review only

		individuals (i.e., all regression coefficients, and model intercept or baseline survival at a given time point).	
Model specification	#15b	If developing a prediction model, explain how to the use it.	NA – not developing a model
Model performance	#16	Report performance measures (with CIs) for the prediction model.	10
Model-updating	#17	If validating a model, report the results from any model updating, if done (i.e., model specification, model performance).	10

Discussion

Limitations	#18	Discuss any limitations of the study (such as nonrepresentative sample, few events per predictor, missing data).	11,12
Interpretation	#19a	For validation, discuss the results with reference to performance in the development data, and any other validation data	12
Interpretation	#19b	Give an overall interpretation of the results, considering objectives, limitations, results from similar studies, and other relevant evidence.	12

For peer review only

Implications	#20	Discuss the potential clinical use of the model and implications for future research	12
**Other information**			
Supplementary information	#21	Provide information about the availability of supplementary resources, such as study protocol, Web calculator, and data sets.	13
Funding	#22	Give the source of funding and the role of the funders for the present study.	3

None The TRIPOD checklist is distributed under the terms of the Creative Commons Attribution License CC-BY. This checklist can be completed online using https://www.goodreports.org/, a tool made by the EQUATOR Network in collaboration with Penelope.ai

For peer review only

BMJ Open

External validation of the QCovid 2 and 3 risk prediction algorithms for risk of COVID-19 hospitalisation and mortality in adults: national cohort study in Scotland

Journal:	*BMJ Open*
Manuscript ID	bmjopen-2023-075958.R1
Article Type:	Original research
Date Submitted by the Author:	04-Sep-2023
Complete List of Authors:	Kerr, Steven; University of Edinburgh<br>Millington, Tristan; The University of Edinburgh Usher Institute of Population Health Sciences and Informatics,<br>Ruden, Igor; The University of Edinburgh<br>McCowan, Colin ; University of St. Andrews<br>Tibble, Holly; University of Edinburgh, Usher Institute of Population Health Sciences and Informatics; Asthma UK Centre for Applied Research,<br>Jeffrey, Karen; University of Edinburgh, Usher Institute; University of Edinburgh,<br>Fagbamigbe, Adeniyi Francis; University of Aberdeen; University of Ibadan, Department of Epidemiology and Medical Statistics<br>Simpson, Colin; Victoria University of Wellington<br>Robertson, Chris; University of Strathclyde, Department of Mathematics and Statistics<br>Hippisley-Cox, Julia; University of Oxford, Nuffield Department of Primary Care Health Sciences<br>Sheikh, Aziz; University of Edinburgh, Division of Community Health Sciences
**Primary Subject Heading**:	Health informatics
Secondary Subject Heading:	Respiratory medicine
Keywords:	Health informatics < BIOTECHNOLOGY & BIOINFORMATICS, RESPIRATORY MEDICINE (see Thoracic Medicine), Epidemiology < INFECTIOUS DISEASES

SCHOLARONE™
Manuscripts

I, the Submitting Author has the right to grant and does grant on behalf of all authors of the Work (as defined in the below author licence), an exclusive licence and/or a non-exclusive licence for contributions from authors who are: i) UK Crown employees; ii) where BMJ has agreed a CC-BY licence shall apply, and/or iii) in accordance with the terms applicable for US Federal Government officers or employees acting as part of their official duties; on a worldwide, perpetual, irrevocable, royalty-free basis to BMJ Publishing Group Ltd ("BMJ") its licensees and where the relevant Journal is co-owned by BMJ to the co-owners of the Journal, to publish the Work in this journal and any other BMJ products and to exploit all rights, as set out in our licence.

The Submitting Author accepts and understands that any supply made under these terms is made by BMJ to the Submitting Author unless you are acting as an employee on behalf of your employer or a postgraduate student of an affiliated institution which is paying any applicable article publishing charge ("APC") for Open Access articles. Where the Submitting Author wishes to make the Work available on an Open Access basis (and intends to pay the relevant APC), the terms of reuse of such Open Access shall be governed by a Creative Commons licence – details of these licences and which Creative Commons licence will apply to this Work are set out in our licence referred to above.

Other than as permitted in any relevant BMJ Author's Self Archiving Policies, I confirm this Work has not been accepted for publication elsewhere, is not being considered for publication elsewhere and does not duplicate material already published. I confirm all authors consent to publication of this Work and authorise the granting of this licence.

For peer review only

External validation of the QCovid 2 and 3 risk prediction algorithms for risk of COVID-19 hospitalisation and mortality in adults: national cohort study in Scotland

Dr Steven Kerr PhD, Usher Institute, The University of Edinburgh, Edinburgh, EH8 9AG, UK

Dr Tristan Millington PhD, Usher Institute, The University of Edinburgh, Edinburgh, EH8 9AG, UK

Prof. Igor Rudan MD, Usher Institute, The University of Edinburgh, Edinburgh, UK

Prof. Colin McCowan, School of Medicine, University of St. Andrews, St Andrews, United Kingdom

Dr Holly Tibble PhD, Usher Institute, The University of Edinburgh, Edinburgh, UK

Dr Karen Jeffrey PhD, Usher Institute, The University of Edinburgh, Edinburgh, UK

Dr Adeniyi Francis Fagbamigbe, PhD, Institute of Applied Health Sciences, University of Aberdeen, AB25 2ZD, Aberdeen, UK; Department of Epidemiology and Medical Statistics, Faculty of Public Health, College of Medicine, University of Ibadan, Ibadan, Nigeria,

Prof. Colin R Simpson PhD, School of Health, Wellington Faculty of Health, Victoria University of Wellington, Wellington, New Zealand and Usher Institute, The University of Edinburgh, Edinburgh, UK

Prof. Chris Robertson PhD, Department of Mathematics and Statistics, University of Strathclyde, Glasgow, UK and Public Health Scotland, Glasgow, UK

Professor Julia Hippisley-Cox MD, Nuffield Department of Primary Care Health Sciences, University of Oxford

Prof. Aziz Sheikh MD, Usher Institute, The University of Edinburgh, Edinburgh, UK

Correspondance to: Dr Steven Kerr PhD, steven.kerr@ed.ac.uk

For peer review only

Word Count:

KEY MESSAGES

What is the key question?

Did the QCovid 2 and 3 algorithms accurately predict the risk of COVID-19 hospitalisation and death in Scotland?

What is the bottom line?

We found good performance predicting COVID-19 hospitalisations and deaths in Scotland. The Harrell's C score was consistently high across all subgroups, but the algorithm overestimated the total number of COVID-19 hospitalisations and deaths.

Why read on?

It is important to validate risk prediction tools in populations outside the data that they were trained on to inform their generalisability and use in policymaking.

For peer review only

ABSTRACT

Objective: The QCovid 2 and 3 algorithms are risk prediction tools developed during the second wave of the COVID-19 pandemic that can be used to predict risk of COVID-19 hospitalisation and mortality, taking vaccination status into account. In this paper we assess their performance in Scotland.

Methods: We used the EAVE II national COVID-19 data platform consisting of individual-level data for the population of Scotland (5.4 million residents). Primary care data were linked to reverse-transcription polymerase chain reaction (RT-PCR) virology testing, hospitalisation and mortality data. We assessed the discrimination and calibration of the QCovid 2 and 3 algorithms in predicting COVID-19 hospitalisations and deaths between 8 December 2020 and 15 June 2021.

Results: Our validation dataset comprised 465,058 individuals, aged 19-100. We found the following performance metrics (95% confidence intervals) for QCovid 2 and 3: Harrell's C 0.84 (0.82, 0.86) for hospitalisation, and 0.92 (0.90, 0.94) for death, observed-expected ratio of 0.24 for hospitalisation and 0.26 for death (i.e. both the number of hospitalisations and the number of deaths were overestimated), and a Brier score of 0.0009 (0.00084, 0.00096) for hospitalisation and 0.00036 (0.00032, 0.0004) for death.

Conclusions: We found good discrimination of the QCovid 2 and 3 algorithms in Scotland, although performance was worse in higher age groups. Both the number of hospitalisations and the number of deaths were overestimated.

Funding: National Institute for Health Research (NIHR) following a commission by the Chief Medical Officer for England. EAVE II is funded by the Medical Research Council (MC_PC_19075) with the support of BREATHE: the Health Data Research Hub for Respiratory Health (MC_PC_19004), which is funded through the UK Research and Innovation Industrial Strategy Challenge Fund and delivered through Health Data Research UK. Additional support has been provided through Public Health Scotland and the Community Health and Social Care Directorate of the Scottish Government.

Keywords: COVID-19, Clinical epidemiology

For peer review only

INTRODUCTION

In December 2019, a novel coronavirus (SARS-CoV-2) emerged in Wuhan, China. The World Health Organization (WHO) declared the outbreak a public health emergency of international concern on 30 January 2020, and then a pandemic on 11 March 2020. The index case in Scotland was identified on 1 March 2020. As of 26 March 2023, there have been over 2.1 million COVID-19 cases in Scotland, with over 17,000 deaths.[1]

Algorithms have been developed to identify people who are at risk of severe COVID-19 outcomes such as hospitalisation and death and guide public health policy.[2-7] The QCovid algorithms are risk-scoring systems that predict the probability of COVID-19 hospitalisation and death. The original QCovid algorithm was commissioned by the Chief Medical Officer (CMO) for England on behalf of the UK Government. QCovid was used by the UK Government to inform policies on shielding and vaccine prioritisation for England,[2] and has been independently and externally validated in England [3] Scotland [4] and Wales [5]. In Scotland, the original QCovid algorithm was found to perform well, with close correspondence between the observed and predicted risks. The Harrell's C scores for hospitalisations and deaths in males and females were 0.809 and 0.946, respectively over the first period of testing (1 March 2020 – 30 April 2020).[4] England [3] and Wales [5] had similar results, finding very good performance.

QCovid was updated at the request of the CMO for England to take account of the evolving nature of the pandemic and vaccination status.[6] This consisted of two algorithms: QCovid 2 which predicted the probability of hospitalisation and death for unvaccinated individuals, and QCovid 3 which predicted the probability of hospitalisation and death for individuals who had received one or two doses of either the ChAdOx1 nCov-19 (Oxford AstraZeneca) or BNT162b2 (Pfizer-BioNTech) vaccines. These algorithms were developed using the QResearch database.[7]

The training cohort for QCovid2 consisted of unvaccinated individuals aged 19 – 100 years, observed between 1 September 2020 and 31 May 2021. The training cohort for QCovid3 consisted of vaccinated individuals aged 19 – 100 years old, observed between 8 December 2020 and 15 June 2021.

Following a request from the Scottish Government, we sought to externally validate these QCovid2 and 3 algorithms for the adult Scottish population. We used a common protocol for validating these algorithms across the four UK nations.[8] We studied the performance of QCovid 2 and 3 between 8 December 2020 and 15 June 2021, the same time period that the algorithm was trained on.

METHODS

The following information largely overlaps with our previous paper [4] validating the first QCovid algorithm, because the methodology was very similar.

Study design

We carried out an external validation of the QCovid 2 and 3 algorithms using the Early Pandemic Evaluation and Enhanced Surveillance of COVID-19 (EAVE II) platform, which contains electronic health records for 5.4 million (~99% of the population) people in Scotland. QCovid 3 accounts for competing risks, whereas QCovid and QCovid 2 do not. Calculating absolute risk of the outcome of interest in a Cox model with competing risks comes with a significant increase in computational demand. Due to limitations on computational resources, we performed this validation using a random 10% sample of the cohort.

Datasets

We had data from all 940 Scottish primary care practices. These were linked to the Electronic Communication of Surveillance in Scotland (ECOSS; national database for all virology testing including NHS and UK Government test centre data), the Scottish Morbidity Record (record of hospitalisation data), and National Records Scotland (death certification) data as part of the EAVE II platform. A more detailed description of the data can be found in our cohort profile in the supplementary material. Table S1 shows a description of the cohort as well as the 10% sample. A data dictionary for the EAVE II project is available online. [9]

Selection criteria

Any individual in the linked dataset aged between 19 and 100 years on 8 December 2020 was eligible for inclusion. Anyone who had a COVID-19 related hospitalisation before the start of follow-up for each dose was excluded from the hospitalisation analysis.

Exposures

For peer review only

Predictor variables were those used in the QCovid 2 and 3 algorithms.[6] These are detailed in the supplementary material in Box S1. All predictor variables were taken as the most recent recorded value in the data at cohort entry.

Outcomes

The primary outcomes were time to COVID-19 hospitalisation, and time to COVID-19 death. COVID-19 hospitalisation was defined as hospitalisation with a positive reverse transcription polymerase chain reaction (RT-PCR) COVID-19 test within 28 days prior to or admission with ICD-10 (International Classification of Disease version 10) codes for COVID-19 (U07.1, U07.2). COVID-19 death was defined as all-cause death within 28 days of a post-positive RT-PCR test, or death with ICD-10 codes for COVID-19 on the death certificate from National Records Scotland.

Missing data

We could not obtain data relating to some conditions used in the QCovid 2 and 3 algorithms. We did not have reliable data available indicating whether the individual had a bone marrow or stem cell transplant in the last six months, whether they had received radiotherapy in the last six months, whether they had been prescribed immunosuppressants, oral steroids or anti-leukotriene or long acting beta2-agonists four or more times in the last six months, whether they had irritable bowel syndrome, and whether they had received a solid organ transplant. The values of these variables were set to "none". We had data on the type of diabetes, but not if it was controlled. If the individual had type 1 diabetes, we took them to be in the uncontrolled category, and if they had type 2 diabetes, we took them to be in the controlled category, as these were the most populous categories in the training data.[6] For all other comorbidities/treatments, a missing value was taken to indicate absence of that comorbidity/treatment.

Reliable ethnicity data were not available, and all individuals were assigned to "White British". In the 2011 Scottish census, 96% of the population was in this ethnic group.[10] The most fine-grained residential location information available in our dataset was data zone, which is a geographical designation comprising of groups of UK Census output areas. Output areas typically consist of ~300 people, whereas data zones typically consist of 500–1,000 people.[11] Townsend Deprivation Scores [12] for each output area were obtained from the 2011 UK census.[11] We took the median value of Townsend Deprivation Scores for the

For peer review only

output areas comprising each data zone to get a deprivation score for each data zone. Missing values for Townsend Deprivation Scores were replaced with the mean value for the cohort. Missing values in the housing category variable were taken to indicate the individual was neither homeless, nor resident in a care home.

We used single imputation by chained equations [12] to impute missing values for body mass index (BMI). There is some evidence of an association between lower levels of socioeconomic status and higher BMI in economically developed countries.[13] Sex is known to be associated with BMI, as is coronary artery disease and diabetes.[14]

There were no missing values for any of the other independent variables.

Model validation

To calculate the risk for those who were unvaccinated, the baseline survival rate was provided on the following days: 0, 30, 60, 90, 183, 210, 240. We linearly interpolated the logarithm of the baseline survival rate to obtain values for intermediate days. We calculated probability of event for an individual either on the day of event, or the day of censoring.

We applied versions 2 and 3 of the QCovid algorithm to a random 10% sample of our cohort, and computed Harrell's Concordance,[15] the Brier score, Royston's D,[16] R^2 [17] and observed-expected (OE) ratio for the period 8 December 2020 to 15 June 2021. As well as calculating these metrics for the entire cohort sample, we also calculated them for several sub-cohorts. These sub-cohorts consisted of unvaccinated individuals, individuals followed up after first and second dose, and the age ranges, 19-64, 65-79 and 80-100.

Harrell's Concordance is a performance metric that characterises the tendency for people with higher hazard rates to have earlier events. It takes values between 0 and 1, with 0 indicating poorer performance and 1 indicating better performance. The Brier score is a measure of forecast accuracy that is equal to the mean squared prediction error in the case of a binary outcome variable. It takes values greater than 0, with zero indicating the best performance. Royston's D is a measure of 'separation' between survival curves. Higher values of Royston's D indicate predicted hazard rates have more discriminative ability. R^2 is a measure of the proportion of variation in survival time explained by the model. It takes values between 0 and 1, with 0 indicating poorer performance and 1 indicating better

For peer review only

performance. We calculated R^2 for survival models as defined in [17]. The OE ratio is the number of observed events divided by the expected number of events predicted by the model. A value of 1 indicates that the total number of events is exactly correctly predicted, and a value less than 1 indicates that the total number of events predicted was greater than observed. We made plots of observed vs expected risk on the day of event/censoring by vigintiles (20 groups) of predicted hazard rate. We also calculated recalibrated risk by scaling predicted risks by a multiplicative constant so that expected total number of events predicted was equal to observed total number of events. We used this recalibrated risk to calculate recalibrated Brier scores and made re-calibrated observed versus expected plots.

Reporting

This study is reported in accordance with the Transparent reporting of a multivariable prediction model for individual prognosis or diagnosis (TRIPOD) guidelines. [18]

Patient and public involvement

The EAVE II Public Advisory Group reviewed the online QCovid tools and the preliminary results from this paper. Although overall in favour of this work, they expressed some concern at the lack of ethnicity data for our results, and that the online tools would need significant editing to be accessible to the publics.

Ethics and permissions

Ethical permission for this study was granted by the South East Scotland Research Ethics Committee 02 [12/SS/0201]. The Public Benefit and Privacy Panel Committee of Public Health Scotland, approved the linkage and analysis of the de–identified datasets for this project [1920-0279]. As we had approvals to use anonymised patient data without consent, there was no pathway for patients to opt out.

RESULTS

In our 10% cohort sample, there were 763 COVID-19 hospitalisations in the validation period, and 393 COVID-19 deaths. Table 1 shows the OE ratio, Harrell's C, Brier score, Royston's D, R^2 and recalibrated Brier score with outcomes of COVID-19 hospitalisation and COVID-19 death for the entire cohort sample, as well as subgroups stratified by follow up after different vaccine doses. Table 2 shows the same statistics, stratified by age group. Figure 1 plots the observed and expected risk of hospitalisation and death by vigintiles of the

For peer review only

hazard rate (HR) for the entire sample, the subset who were unvaccinated and those during follow up after first and second dose, and Figure 2 shows the same set of figures but recalibrated with the actual number of events. The vigintiles were numbered so that higher vigintiles had a higher hazard rate (i.e., those in vigintile 1 were in the lowest hazard rate category, those in vigintile 20 were in the higher hazard rate category).

Vaccine dose

Focusing firstly on hospitalisations stratified by vaccine dose (shown in the top half of Table 1), according to Harrell's C, Royston's D and the R^2 metric, performance was similar across all sub-groups, but better for the entire sample cohort. This is because combining data for unvaccinated with vaccinated people created a large number of additional concordant pairs. Both the Brier score and the recalibrated Brier scores were noticeably worse for the unvaccinated than for those who had one or two vaccine doses, or the entire sample cohort.

In terms of COVID-19 deaths (shown in the bottom half of Table 1), we found that the model performed similarly according to Harrell's C across the vaccine dose follow-up categories. Again, across the other metrics bar the OE ratio, performance was worse for the unvaccinated than for those who had one or two vaccine doses, or for the entire sample cohort.

For both events, the performance according to Harrell's C was very good, and was similar to that in the original study on their validation cohort (0.84 (0.82, 0.86) for hospitalisation in this paper vs 0.85 in the original study, and 0.92 (0.90 – 0.94) in this paper vs 0.93 in the original study) [6]. The D statistic performance in this paper was worse than in the original study (2.21 (2.022, 2.218) vs 2.83 (2.59, 3.08) for hospitalisation and 2.77 (2.61, 2.93) vs 3.46 (3.19, 3.73) for death).

Age

Looking at the results for COVID-19 hospitalisation stratified by age (shown in top half of Table 2), the model performed less well for the older age groups (65 – 79, ≥80) compared to the younger group (18 – 64) according to all metrics, except for the OE ratio. This pattern is also repeated for COVID-19 death stratified by age (shown in the bottom half of Table 2).

Vigintile plots

For peer review only

Figure 1 shows a set of vigintile plots comparing the predicting risk to the observed risk for the full cohort and two sub cohorts – those who were unvaccinated and those with a first and second dose. Figure 2 shows the same set of vigintile plots, but with the risks recalibrated.

The vigintile plots on the left-hand side of Figure 1 show the model's performance in terms of predicting COVID-19 hospitalisations (left hand side) and deaths (right hand size). For both events, the models tended to overestimate the events across all stratifications. The estimates tended to be worse for the higher risk vigintiles.

Figure 2 shows the same plots after we recalibrated the models. After this we found that most of the vigintiles had a good estimation of the number of events after recalibration. Across the stratifications, there was no consistent pattern of over-estimating or underestimating the number of events for specific vigintiles. The predictions for the ≥80 years sub-cohort, particularly for death, were not as good as for the other cohorts. This may have been due to the small sample size combined with the larger risk scores.

DISCUSSION

Statement of principal findings

In this external validation of the QCovid 2 and 3 algorithms in Scotland, we found the algorithms performed well with regards to discrimination as measured by the C statistic. The total number of events was overestimated for hospitalisations and deaths across all subgroups. Performance was broadly similar to that in the original paper.[6]

Strengths and Limitations

Our study had a number of important strengths. As in our previous study, [4] we developed a unique linked dataset covering 99% of the population resident in Scotland. The EAVE II database [19] is one of the few national individual patient-level linked research databases in the world.[20] We evaluated the performance of the QCovid 2 and 3 algorithms according to all metrics used in the original paper [6] and in the common protocol agreed between the four UK nations.[8]

However, our work has several limitations. Firstly, we did not have access to ethnicity data, so all individuals were set to 'White British' ethnicity. We believe modal substitution for ethnicity was reasonable because the most recent Scottish census indicated that 96% of the

For peer review only

residents of Scotland identified their ethnicity as ‘White’.[10] There was significant missingness in the BMI data, with 2,495,504 (55.6%) missing values. We used single imputation by chained equations to impute these missing values for BMI. Multiple imputation was not used due to limitations on computational resources. The most fine-grained residential location information available in our dataset was data zone, which typically consists of multiple 2011 UK census output areas. We took the median value of the Townsend Deprivation Scores for the output areas comprising each data zone to get a deprivation score for each data zone. Missing values of Townsend Deprivation Scores were replaced with the average value for the cohort. Higher levels of deprivation as measured by Townsend Deprivation Scores was associated with increased predicted risk of COVID-19 hospitalisation and death in the QCovid 2 and 3 algorithms. We also did not have data for several clinical risk variables, so individuals were assigned to the category for absence of the condition. This will have had the effect of under-estimating risk in people with these characteristics. We also validated on a 10% sample randomly chosen rather than the full EAVE II cohort due to limitations on computational resources. This led to there being few samples in some of the higher risk groups, and wider confidence intervals on the metrics.

Interpretation

The QCovid 2 and 3 risk prediction algorithms performed well in the Scottish population in the period they were trained for.

Implications for policy, practise and research

Our results indicate that QCovid 2 and 3 would have been appropriate for use as a risk prediction tool for COVID-19 hospitalisation and death in Scotland during our study period. For future research, taking UK-wide perspectives on data availability when developing risk prediction tools should be considered if these tools are to be used nationally.

CONCLUSION

Risk prediction tools are valuable for identifying individuals at the highest risk of experiencing severe outcomes, and can be used by policy makers to help mitigate these risks. However, these tools must be externally validated on cohorts that were not used to train them. We evaluated the QCovid2 and 3 algorithms in Scotland using a variety of metrics and across

For peer review only

several sub-groups. We found good performance overall, and many measures were similar to those in the original paper.

Acknowledgements: The authors would like to thank staff at Public Health Scotland, Albasoft Ltd, the general practices that contributed data to the study and the EAVE II Collaborators. AS, JM and CR serve on The Scottish Government's COVID-19 Chief Medical Officer's Advisory Group and the New and Emerging Respiratory Virus Threats Advisory Group (NERVTAG) Risk Stratification Subgroup.

Data sharing: All code, metadata and documentation for this project is publicly available at https://github.com/EAVE-II/QCovid2-3-validation. A data dictionary is available at https://github.com/EAVE-II/EAVE-II-data-dictionary. Most of the data that were used in this study are highly sensitive and will not be made available publicly.

Authors and contributors: AS and JHC conceptualised the study. SK carried out the formal analysis. TM wrote an initial draft of the manuscript. All authors assisted with review and editing. CR and TM have verified the underlying data.

Declaration of interest: Dr. Hippisley-Cox reports grants from MRC, grants from Wellcome Trust, grants from NIHR, during the conduct of the study; JHC is a founder and shareholder of ClinRisk ltd and was its medical director until 31st May 2019. ClinRisk Ltd produces open and closed source software to implement clinical risk algorithms (outside this work) into clinical computer systems. JHC was chair of the NERVTAG risk stratification subgroup and a member of SAGE COVID-19 groups and the NHS group advising on prioritisation of use of monoclonal antibodies in COVID-19 infection. Dr. Sheikh reports grants from NIHR, grants from MRC, grants from HDR UK, during the conduct of the study. All other authors report no conflict of interest.

For peer review only

406 **Table 1:** Performance metrics (95% confidence intervals) stratified by vaccination status for
407 COVID-19 hospitalisation and death.

Group	Observed-expected ratio	C	Brier Score	Royston's D	Recalibrated Brier Score	R^2
**Hospitalisation**						
Everyone	0.24	0.84 (0.82 - 0.86)	0.0009 (0.00084 - 0.00096)	2.12 (2.022 - 2.218)	0.00091 (0.00085 - 0.00097)	0.52 (0.49 - 0.54)
Unvaccinated	0.23	0.73 (0.71 - 0.75)	0.00209 (0.00193 - 0.00225)	1.33 (1.2124 - 1.4476)	0.00211 (0.00195 - 0.00227)	0.3 (0.26 - 0.33)
First and second dose vaccinated	0.33	0.72 (0.66 - 0.78)	0.00015 (0.00013 - 0.00017)	1.43 (1.136 - 1.724)	0.00015 (0.00013 - 0.00017)	0.33 (0.23 - 0.42)
**Death**						
Total population	0.26	0.92 (0.9 - 0.94)	0.00036 (0.00032 – 0.004)	2.77 (2.6132 - 2.9268)	0.00036 (0.00032 – 0.0004)	0.65 (0.62 - 0.67)
Unvaccinated	0.34	0.88 (0.86 - 0.9)	0.00077 (0.00069 - 0.00085)	2.35 (2.1736 - 2.5264)	0.00077 (0.00069 - 0.00085)	0.57 (0.53 - 0.6)

For peer review only

First and second dose vaccinated	0.13	0.94 (0.92 - 0.96)	0.00001 (0.00008 - 0.00012)	3.09 (2.7372 - 3.4428)	1.0001 (0.00008 - 0.00012)	0.7 (0.64 - 0.74)

Table 2: Performance metrics (95% confidence intervals) for COVID-19 hospitalisation and death stratified by age group.

Age Group	Observed -expected ratio	C	Brier Score	Royston's D	Recalibrated Brier Score	R^2
**Hospitalisation**						
Age 18-64	0.21	0.82 (0.8 - 0.84)	0.00067 (0.00061 - 0.00073)	1.9 (1.7628 - 2.0372)	0.00068 (0.00062 - 0.00074)	0.46 (0.42 - 0.5)
Age 65-79	0.29	0.80 (0.78 - 0.82)	0.00112 (0.00098 - 0.00126)	1.70 (1.504 - 1.896)	0.00113 (0.00099 - 0.00127)	0.41 (0.35 - 0.46)
Age 80+	0.27	0.78 (0.76 - 0.8)	0.00255 (0.0022 - 0.0029)	1.55 (1.3344 - 1.7656)	0.00258 (0.00223 - 0.00293)	0.36 (0.3 - 0.43)
**Death**						
Age 18-64	0.15	0.85 (0.81 - 0.89)	0.00007 (0.00005 – 0.00009)	1.96 (1.5288 - 2.3912)	0.00007 (0.00005 – 0.00009)	0.48 (0.36 - 0.58)
Age 65-79	0.28	0.82 (0.8 - 0.84)	0.00052 (0.00042 - 0.00062)	1.77 (1.476 - 2.064)	0.00052 (0.00042 - 0.00062)	0.43 (0.34 - 0.51)
Age 80+	0.3	0.73 (0.71	0.00309 (0.0027 - 0.00348)	1.23 (1.034 - 1.426)	0.00311 (0.0027 - 0.00352)	0.26 (0.2 - 0.33)

For peer review only

		- 0.75)				

Figure 1: Vigintile plots of expected vs observed risks for the cohort and a selection of sub-cohorts.

Figure 2: Vigintile plots of expected vs observed recalibrated risks for the cohort and a selection of sub-cohorts.

References

1. *Deaths involving coronavirus (COVID-19) in Scotland*. 2023; Available from: https://www.nrscotland.gov.uk/statistics-and-data/statistics/statistics-by-theme/vital-events/general-publications/weekly-and-monthly-data-on-births-and-deaths/deaths-involving-coronavirus-covid-19-in-scotland
2. *New technology to help identify those at high risk from COVID-19* [cited 2023; Available from: https://www.gov.uk/government/news/new-technology-to-help-identify-those-at-high-risk-from-covid-19.
3. Nafilyan, V., et al., *An external validation of the QCovid risk prediction algorithm for risk of mortality from COVID-19 in adults: a national validation cohort study in England.* The Lancet Digital Health, 2021. **3**(7): p. e425-e433.
4. Simpson, C.R., et al., *External validation of the QCovid risk prediction algorithm for risk of COVID-19 hospitalisation and mortality in adults: national validation cohort study in Scotland.* Thorax, 2022. **77**(5): p. 497-504.
5. Lyons, J., et al., *Validating the QCOVID risk prediction algorithm for risk of mortality from COVID-19 in the adult population in Wales, UK.* Int J Popul Data Sci, 2020. **5**(4): p. 1697.
6. Hippisley-Cox, J., et al., *Risk prediction of covid-19 related death and hospital admission in adults after covid-19 vaccination: national prospective cohort study.* BMJ, 2021. **374**: p. n2244.
7. Hippisley-Cox, J., D. Stables, and M. Pringle, *QRESEARCH: a new general practice database for research.* Inform Prim Care, 2004. **12**(1): p. 49-50.
8. Kerr, S., et al., *Common protocol for validation of the QCOVID algorithm across the four UK nations.* BMJ Open, 2022. **12**(6): p. e050994.
9. *EAVE II Data Dictionary*, E.I. Analysts, Editor. 2021.
10. *Scotland's Census - Ethnicity*. 2021; Available from: https://www.scotlandscensus.gov.uk/census-results/at-a-glance/ethnicity/.
11. Service., O.f.N.S.N.R.o.S.N.I.S.a.R.A.U.D. *2011 UK Census Townsend Deprivation Scores*. 2011 [cited 2023; Available from: https://statistics.ukdataservice.ac.uk/dataset/2011-uk-townsend-deprivation-scores.

For peer review only

12. Little, R.J. and D.B. Rubin, *Single imputation methods.* Statistical analysis with missing data, 2002: p. 59-74.
13. McLaren, L., *Socioeconomic status and obesity.* Epidemiologic reviews, 2007. **29**(1): p. 29-48.
14. Bays, H.E., R.H. Chapman, and S. Grandy, *The relationship of body mass index to diabetes mellitus, hypertension and dyslipidaemia: comparison of data from two national surveys.* Int J Clin Pract, 2007. **61**(5): p. 737-47.
15. Harrell, F.E., Jr., et al., *Evaluating the yield of medical tests.* Jama, 1982. **247**(18): p. 2543-6.
16. Royston, P. and W. Sauerbrei, *A new measure of prognostic separation in survival data.* Stat Med, 2004. **23**(5): p. 723-48.
17. Royston, P., *Explained variation for survival models.* The Stata Journal, 2006. **6**(1): p. 83-96.
18. Moons, K.G., et al., *Transparent Reporting of a multivariable prediction model for Individual Prognosis or Diagnosis (TRIPOD): explanation and elaboration.* Ann Intern Med, 2015. **162**(1): p. W1-73.
19. Simpson, C.R., et al., *Early Pandemic Evaluation and Enhanced Surveillance of COVID-19 (EAVE II): protocol for an observational study using linked Scottish national data.* BMJ Open, 2020. **10**(6): p. e039097.
20. Simpson, C.R., et al., *The UK's pandemic influenza research portfolio: a model for future research on emerging infections.* Lancet Infect Dis, 2019. **19**(8): p. e295-e300.

For peer review only

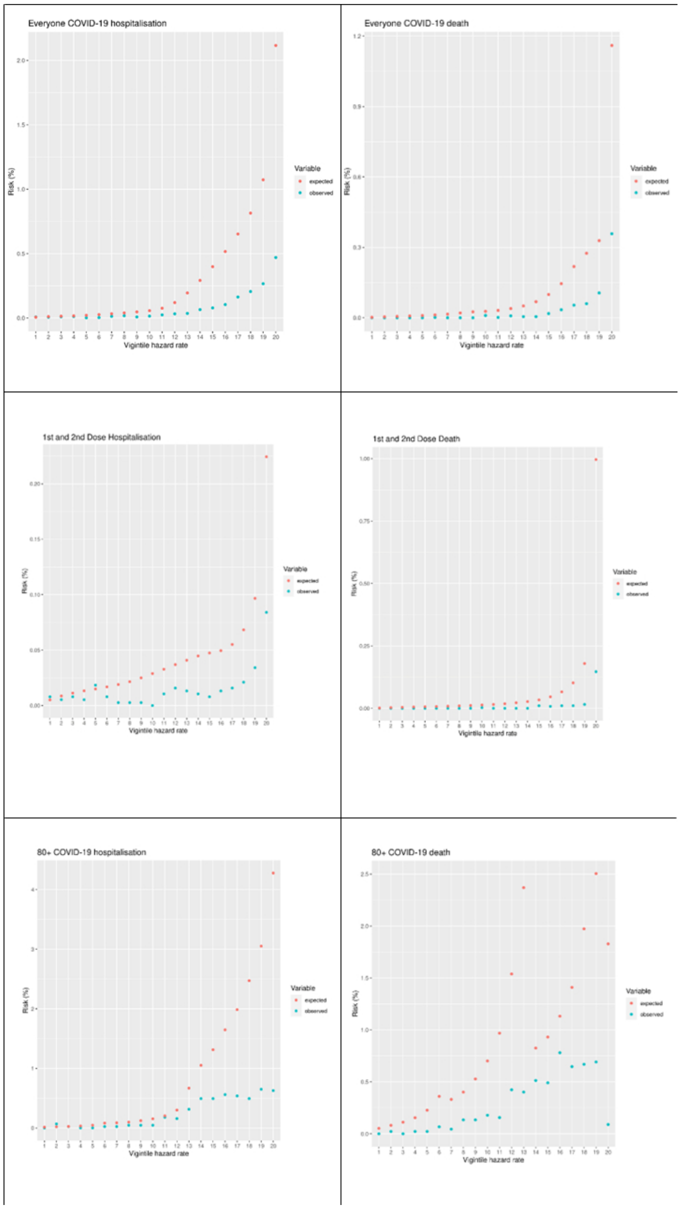

Figure 1: Vigintile plots of expected vs observed risks for the cohort and a selection of sub-cohorts.

481x855mm (38 x 38 DPI)

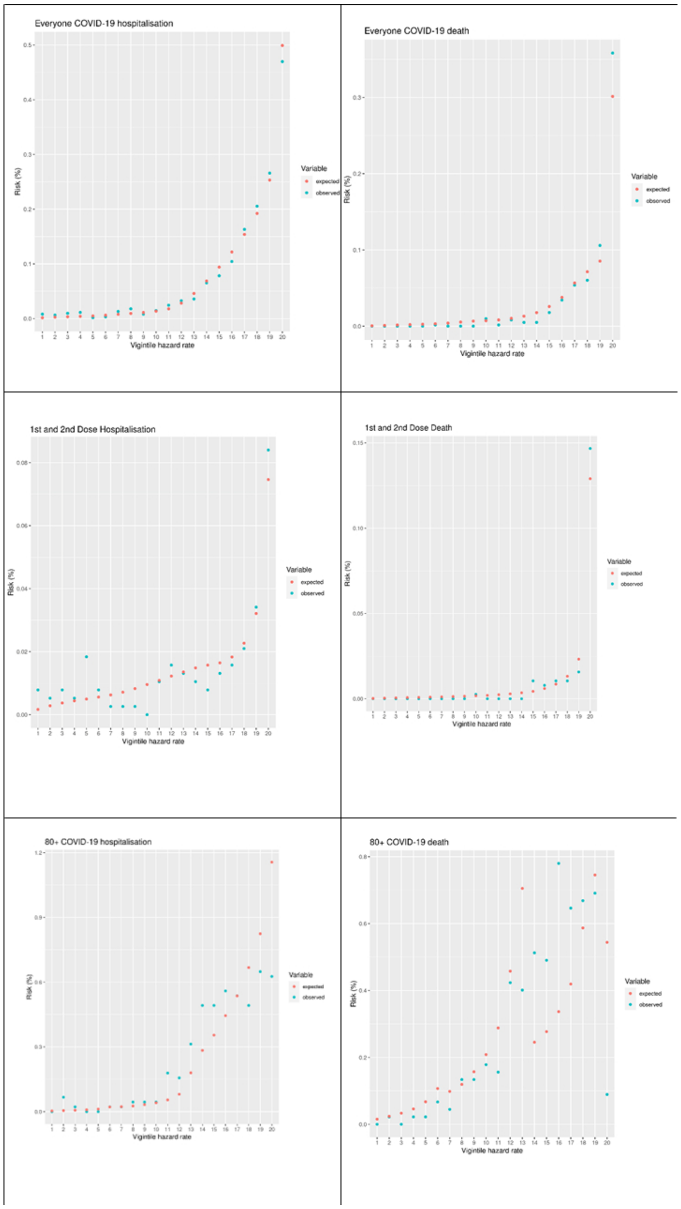

Figure 2: Vigintile plots of expected vs observed recalibrated risks for the cohort and a selection of sub-cohorts.

481x855mm (38 x 38 DPI)

Table S1: Summary statistics for the full cohort and 10% sample

Characteristics	Levels	Entire cohort	10% cohort sample
Total		4650585 (100.0)	465058 (100.0)
COVID-19 Deaths		4049 (0.1)	393 (0.1)
COVID-19 Hospitalisations		9547 (0.2)	763 (0.2)
Sex	Female	2358990 (50.7)	235821 (50.7)
	Male	2291595 (49.3)	229237 (49.3)
Age	Mean (SD)	50.3 (18.8)	50.3 (18.9)
Age group	19-39	1581219 (34.0)	158052 (34.0)
	40-59	1533605 (33.0)	152985 (32.9)
	60-74	978827 (21.0)	98190 (21.1)
	75-100	556934 (12.0)	55831 (12.0)
SIMD quintile	1 - Most deprived	925925 (19.9)	92546 (19.9)
	2	918884 (19.8)	92073 (19.8)
	3	918091 (19.7)	91868 (19.8)
	4	921838 (19.8)	92219 (19.8)
	5 - Least deprived	924963 (19.9)	92392 (19.9)
	(Missing)	40884 (0.9)	3960 (0.9)
Urban rural classification	Large Urban Areas	1671719 (35.9)	166887 (35.9)
	Other Urban Areas	1652152 (35.5)	165274 (35.5)
	Accessible Small Towns	424031 (9.1)	42739 (9.2)
	Remote Small Towns	221513 (4.8)	22271 (4.8)
	Accessible Rural	413436 (8.9)	41170 (8.9)
	Remote Rural	226850 (4.9)	22757 (4.9)
	(Missing)	40884 (0.9)	3960 (0.9)
Ethnicity	Asian	102490 (2.2)	10250 (2.2)
	Black	25123 (0.5)	2567 (0.6)
	Mixed	21139 (0.5)	2148 (0.5)

For peer review only

	Other	23584 (0.5)	2383 (0.5)
	Unknown	1562888 (33.6)	156408 (33.6)
	White	2915361 (62.7)	291302 (62.6)
Number of risk groups	0	2725853 (58.6)	272930 (58.7)
	1	1249848 (26.9)	125016 (26.9)
	2	434728 (9.3)	43153 (9.3)
	3 - 4	210691 (4.5)	21024 (4.5)
	5+	29465 (0.6)	2935 (0.6)
BMI	15-18.5	48651 (1.0)	4772 (1.0)
	18.5-25	543001 (11.7)	54377 (11.7)
	25-30	674760 (14.5)	67921 (14.6)
	30-35	431788 (9.3)	43170 (9.3)
	35-40	198273 (4.3)	19835 (4.3)
	40+	133917 (2.9)	13226 (2.8)
	(Missing)	2620195 (56.3)	261757 (56.3)
Housing category	Neither	4624584 (99.4)	462533 (99.5)
	Care home	25217 (0.5)	2457 (0.5)
	Homeless	784 (0.0)	68 (0.0)
Learning disability/Down's syndrome	Neither	4600228 (98.9)	460016 (98.9)
	Down's syndrome	1526 (0.0)	144 (0.0)
	Learning disability	48831 (1.0)	4898 (1.1)
Chronic Kidney Disease	No CKD	4484131 (96.4)	448600 (96.5)
	CKD 3	166431 (3.6)	16455 (3.5)
	CKD 4	19 (0.0)	*
	CKD 5	4 (0.0)	*
Atrial Fibrillation		114239 (2.5)	11502 (2.5)
Asthma		494549 (10.6)	49499 (10.6)
Blood cancer		20162 (0.4)	2049 (0.4)

For peer review only

Congestive Cardiac Failure		46961 (1.0)	4748 (1.0)
Cerebral Palsy		5521 (0.1)	529 (0.1)
Coronary heart disease		191035 (4.1)	19132 (4.1)
Liver cirrhosis		10882 (0.2)	1116 (0.2)
Congenital heart disease		18969 (0.4)	1854 (0.4)
COPD		118884 (2.6)	11922 (2.6)
Dementia		23258 (0.5)	2202 (0.5)
Diabetes Type 1		65238 (1.4)	6434 (1.4)
Diabetes Type 2		205608 (4.4)	20446 (4.4)
Epilepsy		4032 (0.1)	386 (0.1)
Hip, wrist, spine, humerus fracture		1638 (0.0)	140 (0.0)
HIV/AIDS		18937 (0.4)	1865 (0.4)
Severe combined immunodeficiency syndrome		9707 (0.2)	987 (0.2)
Neurological conditions		6916 (0.1)	668 (0.1)
Parkinsons disease		23496 (0.5)	2367 (0.5)
Pulmonary hypertension		11937 (0.3)	1212 (0.3)
Cystic fibrosis, bronchiectasis or alveolitis		88090 (1.9)	8852 (1.9)
Peripheral vascular disease		5452 (0.1)	538 (0.1)
SLE or rheumatoid arthritis		19750 (0.4)	1892 (0.4)
Lung, oral cancer		205 (0.0)	8 (0.0)

For peer review only

Severe mental illness		115619 (2.5)	11431 (2.5)
Sickle cell disease or combined immune deficiency syndrome		41948 (0.9)	4198 (0.9)
Stroke, transient ischaemic attack		22733 (0.5)	2309 (0.5)
Venous thromboembolism		265285 (5.7)	26697 (5.7)

For peer review only

Box S1: Predictor variables in the QCovid 3 algorithm

• Vaccine dose
• Age in years (continuous)
• Sex
• Ethnicity in ten categories (Bangladeshi, Black African, Black Caribbean, Chinese, Indian, Mixed, Pakistani, White British, White other, Other, unknown)
• Townsend deprivation score (continuous)
• Body Mass Index (kg/m2)
• Accommodation (Neither homeless nor care home, care home or nursing home)
• Chronic kidney disease (CKD) – (no CKD, CKD3, CKD4, CKD5, unknown)
• Learning disability (No learning disability, Down’s Syndrome, other learning disability)
• Chemotherapy in last 12 months (none, Chemotherapy group A, B, C, unknown)
• Blood Cancer
• Bone Marrow transplant in the past 6 months
• Rare Lung Diseases (cystic fibrosis, bronchiectasis, or alveolitis)
• Respiratory cancer
• Radiotherapy in last 6 months
• Solid organ transplant
• Prescribed immunosuppressant medication by GP
• Prescribed leukotriene or long-acting beta blockers
• Prescribed regular prednisolone
• Sickle cell disease
• Diabetes
• Chronic obstructive pulmonary disease (COPD)
• Asthma
• Rare pulmonary diseases
• Pulmonary hypertension or pulmonary fibrosis
• Coronary heart disease
• Stroke
• Atrial Fibrillation
• Congestive cardiac failure
• Venous thromboembolism
• Peripheral vascular disease
• Congenital heart disease
• Dementia
• Heart Failure
• Parkinson's disease
• Epilepsy
• Rare neurological conditions
• Cerebral palsy
• Severe mental illness (bipolar disorder, schizophrenia, severe depression)
• Osteoporotic fracture
• Rheumatoid arthritis or Systemic lupus erythematosus
• Cirrhosis of the liver
• Inflammatory Bowel Disease
• HIV/AIDS
• Severe Combined Immunodeficiency
• Previous Positive SARS-CoV-2 positive test result
• Background rate of RT-PCR positivity per 100,000 people

For peer review only

Reporting checklist for prediction model development/validation.

Based on the TRIPOD guidelines.

Instructions to authors

Complete this checklist by entering the page numbers from your manuscript where readers will find each of the items listed below.

Your article may not currently address all the items on the checklist. Please modify your text to include the missing information. If you are certain that an item does not apply, please write "n/a" and provide a short explanation.

Upload your completed checklist as an extra file when you submit to a journal.

In your methods section, say that you used the TRIPOD reporting guidelines, and cite them as:

Collins GS, Reitsma JB, Altman DG, Moons KG. Transparent reporting of a multivariable prediction model for individual prognosis or diagnosis (TRIPOD): The TRIPOD statement.

For peer review only

		Reporting Item	Page Number
**Title**			
	#1	Identify the study as developing and / or validating a multivariable prediction model, the target population, and the outcome to be predicted.	1
**Abstract**			

	#2	Provide a summary of objectives, study design, setting, participants, sample size, predictors, outcome, statistical analysis, results, and conclusions.	3
**Introduction**			
	#3a	Explain the medical context (including whether diagnostic or prognostic) and rationale for developing or validating the multivariable prediction model, including references to existing models.	5
	#3b	Specify the objectives, including whether the study describes the development or validation of the model or both.	6
**Methods**			
Source of data	#4a	Describe the study design or source of data (e.g., randomized trial, cohort, or registry data), separately for the development and validation data sets, if applicable.	6
Source of data	#4b	Specify the key study dates, including start of accrual; end of accrual; and, if applicable, end of follow-up.	6, 8
Participants	#5a	Specify key elements of the study setting (e.g., primary care, secondary care, general	6

For peer review only

		population) including number and location of centres.	
Participants	#5b	Describe eligibility criteria for participants.	6
Participants	#5c	Give details of treatments received, if relevant	NA
Outcome	#6a	Clearly define the outcome that is predicted by the prediction model, including how and when assessed.	7
Outcome	#6b	Report any actions to blind assessment of the outcome to be predicted.	No actions necessary
Predictors	#7a	Clearly define all predictors used in developing or validating the multivariable prediction model, including how and when they were measured	Supplementary material 4
Predictors	#7b	Report any actions to blind assessment of predictors for the outcome and other predictors.	No actions necessary
Sample size	#8	Explain how the study size was arrived at.	6
Missing data	#9	Describe how missing data were handled (e.g., complete-case analysis, single imputation, multiple imputation) with details of any imputation method.	7

For peer review only

Statistical analysis methods	#10a	If you are developing a prediction model describe how predictors were handled in the analyses.	NA – not developing a model
Statistical analysis methods	#10b	If you are developing a prediction model, specify type of model, all model-building procedures (including any predictor selection), and method for internal validation.	NA – not developing a model
Statistical analysis methods	#10c	If you are validating a prediction model, describe how the predictions were calculated.	8
Statistical analysis methods	#10d	Specify all measures used to assess model performance and, if relevant, to compare multiple models.	8
Statistical analysis methods	#10e	If you are validating a prediction model, describe any model updating (e.g., recalibration) arising from the validation, if done	9
Risk groups	#11	Provide details on how risk groups were created, if done.	6
Development vs. validation	#12	For validation, identify any differences from the development data in setting, eligibility criteria, outcome, and predictors.	2

For peer review only

Results

Participants	#13a	Describe the flow of participants through the study, including the number of participants with and without the outcome and, if applicable, a summary of the follow-up time. A diagram may be helpful.	
Participants	#13b	Describe the characteristics of the participants (basic demographics, clinical features, available predictors), including the number of participants with missing data for predictors and outcome.	Supplementary material p1
Participants	#13c	For validation, show a comparison with the development data of the distribution of important variables (demographics, predictors and outcome).	NA – we are validating an external algorithm trained on external data we do not have access to
Model development	#14a	If developing a model, specify the number of participants and outcome events in each analysis.	NA – not developing a model
Model development	#14b	If developing a model, report the unadjusted association, if calculated between each candidate predictor and outcome.	NA – not developing a model
Model specification	#15a	If developing a model, present the full prediction model to allow predictions for	NA – not developing a model

For peer review only

		individuals (i.e., all regression coefficients, and model intercept or baseline survival at a given time point).	
Model specification	#15b	If developing a prediction model, explain how to the use it.	NA – not developing a model
Model performance	#16	Report performance measures (with CIs) for the prediction model.	10
Model-updating	#17	If validating a model, report the results from any model updating, if done (i.e., model specification, model performance).	10
**Discussion**			
Limitations	#18	Discuss any limitations of the study (such as nonrepresentative sample, few events per predictor, missing data).	11,12
Interpretation	#19a	For validation, discuss the results with reference to performance in the development data, and any other validation data	12
Interpretation	#19b	Give an overall interpretation of the results, considering objectives, limitations, results from similar studies, and other relevant evidence.	12

For peer review only

Implications	#20	Discuss the potential clinical use of the model and implications for future research	12
**Other information**			
Supplementary information	#21	Provide information about the availability of supplementary resources, such as study protocol, Web calculator, and data sets.	13
Funding	#22	Give the source of funding and the role of the funders for the present study.	3

None The TRIPOD checklist is distributed under the terms of the Creative Commons Attribution License CC-BY. This checklist can be completed online using https://www.goodreports.org/, a tool made by the EQUATOR Network in collaboration with Penelope.ai

For peer review only

BMJ Open

External validation of the QCovid 2 and 3 risk prediction algorithms for risk of COVID-19 hospitalisation and mortality in adults: a national cohort study in Scotland

Journal:	*BMJ Open*
Manuscript ID	bmjopen-2023-075958.R2
Article Type:	Original research
Date Submitted by the Author:	19-Oct-2023
Complete List of Authors:	Kerr, Steven; The University of Edinburgh Usher Institute of Population Health Sciences and Informatics<br>Millington, Tristan; The University of Edinburgh Usher Institute of Population Health Sciences and Informatics,<br>Ruden, Igor; The University of Edinburgh<br>McCowan, Colin ; University of St. Andrews<br>Tibble, Holly; University of Edinburgh, Usher Institute of Population Health Sciences and Informatics; Asthma UK Centre for Applied Research,<br>Jeffrey, Karen; University of Edinburgh, Usher Institute; University of Edinburgh,<br>Fagbamigbe, Adeniyi Francis; University of Aberdeen; University of Ibadan, Department of Epidemiology and Medical Statistics<br>Simpson, Colin; Victoria University of Wellington<br>Robertson, Chris; University of Strathclyde, Department of Mathematics and Statistics<br>Hippisley-Cox, Julia; University of Oxford, Nuffield Department of Primary Care Health Sciences<br>Sheikh, Aziz; University of Edinburgh, Division of Community Health Sciences
**Primary Subject Heading**:	Health informatics
Secondary Subject Heading:	Respiratory medicine
Keywords:	Health informatics < BIOTECHNOLOGY & BIOINFORMATICS, RESPIRATORY MEDICINE (see Thoracic Medicine), Epidemiology < INFECTIOUS DISEASES

SCHOLARONE™
Manuscripts

I, the Submitting Author has the right to grant and does grant on behalf of all authors of the Work (as defined in the below author licence), an exclusive licence and/or a non-exclusive licence for contributions from authors who are: i) UK Crown employees; ii) where BMJ has agreed a CC-BY licence shall apply, and/or iii) in accordance with the terms applicable for US Federal Government officers or employees acting as part of their official duties; on a worldwide, perpetual, irrevocable, royalty-free basis to BMJ Publishing Group Ltd ("BMJ") its licensees and where the relevant Journal is co-owned by BMJ to the co-owners of the Journal, to publish the Work in this journal and any other BMJ products and to exploit all rights, as set out in our licence.

The Submitting Author accepts and understands that any supply made under these terms is made by BMJ to the Submitting Author unless you are acting as an employee on behalf of your employer or a postgraduate student of an affiliated institution which is paying any applicable article publishing charge ("APC") for Open Access articles. Where the Submitting Author wishes to make the Work available on an Open Access basis (and intends to pay the relevant APC), the terms of reuse of such Open Access shall be governed by a Creative Commons licence – details of these licences and which Creative Commons licence will apply to this Work are set out in our licence referred to above.

Other than as permitted in any relevant BMJ Author's Self Archiving Policies, I confirm this Work has not been accepted for publication elsewhere, is not being considered for publication elsewhere and does not duplicate material already published. I confirm all authors consent to publication of this Work and authorise the granting of this licence.

For peer review only

External validation of the QCovid 2 and 3 risk prediction algorithms for risk of COVID-19 hospitalisation and mortality in adults: a national cohort study in Scotland

Dr Steven Kerr PhD, Usher Institute, University of Edinburgh, Edinburgh, UK

Dr Tristan Millington PhD, Usher Institute, University of Edinburgh, Edinburgh, UK

Prof Igor Rudan MD, Usher Institute, University of Edinburgh, Edinburgh, UK

Prof Colin McCowan, School of Medicine, University of St Andrews, St Andrews, UK

Dr Holly Tibble PhD, Usher Institute, University of Edinburgh, Edinburgh, UK

Dr Karen Jeffrey PhD, Usher Institute, University of Edinburgh, Edinburgh, UK

Dr Adeniyi Francis Fagbamigbe, PhD, Institute of Applied Health Sciences, University of Aberdeen, Aberdeen, UK; and Department of Epidemiology and Medical Statistics, Faculty of Public Health, College of Medicine, University of Ibadan, Ibadan, Nigeria

Prof Colin R Simpson PhD, School of Health, Wellington Faculty of Health, Victoria University of Wellington, Wellington, New Zealand; and Usher Institute, University of Edinburgh, Edinburgh, UK

Prof Chris Robertson PhD, Department of Mathematics and Statistics, University of Strathclyde, Glasgow, UK; and Public Health Scotland, Glasgow, UK

Prof Julia Hippisley-Cox MD, Nuffield Department of Primary Care Health Sciences, University of Oxford, Oxford, UK

Prof Aziz Sheikh MD, Usher Institute, University of Edinburgh, Edinburgh, UK

Correspondence to:
Dr Tristan Millington PhD

For peer review only

Health Informatics Centre, Division of Population and Health Genomics, School of Medicine, University of Dundee, Dundee, United Kingdom, DD1 9SY
tmillington001@dundee.ac.uk/tristan.millington@ed.ac.uk

ABSTRACT

Objective: The QCovid 2 and 3 algorithms are risk prediction tools developed during the second wave of the COVID-19 pandemic that can be used to predict risk of COVID-19 hospitalisation and mortality, taking vaccination status into account. In this study we assess their performance in Scotland.

Methods: We used the EAVE II national COVID-19 data platform consisting of individual-level data for the population of Scotland (5.4 million residents). Primary care data were linked to reverse-transcription polymerase chain reaction (RT-PCR) virology testing, hospitalisation and mortality data. We assessed the discrimination and calibration of the QCovid 2 and 3 algorithms in predicting COVID-19 hospitalisations and deaths between 8 December 2020 and 15 June 2021.

Results: Our validation dataset comprised 465,058 individuals, aged 19-100. We found the following performance metrics (95% confidence intervals) for QCovid 2 and 3: Harrell's C 0.84 (0.82, 0.86) for hospitalisation, and 0.92 (0.90, 0.94) for death, observed-expected ratio of 0.24 for hospitalisation and 0.26 for death (i.e. both the number of hospitalisations and the number of deaths were overestimated), and a Brier score of 0.0009 (0.00084, 0.00096) for hospitalisation and 0.00036 (0.00032, 0.0004) for death.

Conclusions: We found good discrimination of the QCovid 2 and 3 algorithms in Scotland, although performance was worse in higher age groups. Both the number of hospitalisations and the number of deaths were overestimated.

Keywords: COVID-19, Clinical epidemiology

Strengths and limitations of this study

For peer review only

- We used a population-level dataset to perform an external validation of the QCovid 2 and 3 algorithms.
- We validated these algorithms on the same time interval as in the original derivation study.
- We only used a 10% sample of the population due to computational issues.
- Some data used in the algorithms was missing.

For peer review only

INTRODUCTION

In December 2019, a novel coronavirus (SARS-CoV-2) emerged in Wuhan, China. The World Health Organization (WHO) declared the outbreak a public health emergency of international concern on 30 January 2020, and then a pandemic on 11 March 2020. The index case in Scotland was identified on 1 March 2020. As of 26 March 2023, there have been over 2.1 million COVID-19 cases in Scotland, with over 17,000 deaths.[1]

Algorithms have been developed to identify people who are at risk of severe COVID-19 outcomes such as hospitalisation and death and guide public health policy.[2-7] The QCovid algorithms are risk-scoring systems that predict the probability of COVID-19 hospitalisation and death. The original QCovid algorithm was commissioned by the Chief Medical Officer (CMO) for England on behalf of the UK Government. QCovid was used by the UK Government to inform policies on shielding and vaccine prioritisation for England,[2] and has been independently and externally validated in England [3] Scotland [4] and Wales [5]. In Scotland, the original QCovid algorithm was found to perform well, with close correspondence between the observed and predicted risks. The Harrell's C scores for hospitalisations and deaths in males and females were 0.809 and 0.946, respectively over the first period of testing (1 March 2020 – 30 April 2020).[4] England [3] and Wales [5] had similar results, finding very good performance.

QCovid was updated at the request of the CMO for England to take account of the evolving nature of the pandemic and vaccination status.[6] This consisted of two algorithms: QCovid 2 which predicted the probability of hospitalisation and death for unvaccinated individuals, and QCovid 3 which predicted the probability of hospitalisation and death for individuals who had received one or two doses of either the ChAdOx1 nCov-19 (Oxford AstraZeneca) or BNT162b2 (Pfizer-BioNTech) vaccines. These algorithms were developed using the QResearch database.[7]

The training cohort for QCovid2 consisted of unvaccinated individuals aged 19 – 100 years, observed between 1 September 2020 and 31 May 2021. The training cohort for QCovid3 consisted of vaccinated individuals aged 19 – 100 years old, observed between 8 December 2020 and 15 June 2021.

For peer review only

Following a request from the Scottish Government, we sought to externally validate these QCovid2 and 3 algorithms for the adult Scottish population. We used a common protocol for validating these algorithms across the four UK nations.[8] We studied the performance of QCovid 2 and 3 between 8 December 2020 and 15 June 2021, the same time period that the algorithm was trained on.

METHODS

The following information largely overlaps with our previous report [4] validating the first QCovid algorithm, because the methodology was very similar.

Study design

We carried out an external validation of the QCovid 2 and 3 algorithms using the Early Pandemic Evaluation and Enhanced Surveillance of COVID-19 (EAVE II) platform, which contains electronic health records for 5.4 million (~99% of the population) people in Scotland. QCovid 3 accounts for competing risks, whereas QCovid and QCovid 2 do not. Calculating absolute risk of the outcome of interest in a Cox model with competing risks comes with a significant increase in computational demand. Due to limitations on computational resources, we performed this validation using a random 10% sample of the cohort.

Datasets

We had data from all 940 Scottish primary care practices. These were linked to the Electronic Communication of Surveillance in Scotland (ECOSS; national database for all virology testing including NHS and UK Government test centre data), the Scottish Morbidity Record (record of hospitalisation data), and National Records Scotland (death certification) data as part of the EAVE II platform. A more detailed description of the data can be found in our cohort profile in the supplementary material. Table S1 shows a description of the cohort as well as the 10% sample. A data dictionary for the EAVE II project is available online. [9]

Selection criteria

Any individual in the linked dataset aged between 19 and 100 years on 8 December 2020 was eligible for inclusion. Anyone who had a COVID-19 related hospitalisation before the start of follow-up for each dose was excluded from the hospitalisation analysis.

Exposures

For peer review only

Predictor variables were those used in the QCovid 2 and 3 algorithms.[6] These are detailed in the supplementary material in Box S1. All predictor variables were taken as the most recent recorded value in the data at cohort entry.

Outcomes

The primary outcomes were time to COVID-19 hospitalisation, and time to COVID-19 death. COVID-19 hospitalisation was defined as hospitalisation with a positive reverse transcription polymerase chain reaction (RT-PCR) COVID-19 test within 28 days prior to or admission with ICD-10 (International Classification of Disease version 10) codes for COVID-19 (U07.1, U07.2). COVID-19 death was defined as all-cause death within 28 days of a post-positive RT-PCR test, or death with ICD-10 codes for COVID-19 on the death certificate from National Records Scotland.

Missing data

We could not obtain data relating to some conditions used in the QCovid 2 and 3 algorithms. We did not have reliable data available indicating whether the individual had a bone marrow or stem cell transplant in the last six months, whether they had received radiotherapy in the last six months, whether they had been prescribed immunosuppressants, oral steroids or anti-leukotriene or long acting beta2-agonists four or more times in the last six months, whether they had irritable bowel syndrome, and whether they had received a solid organ transplant. The values of these variables were set to "none". We had data on the type of diabetes, but not if it was controlled. If the individual had type 1 diabetes, we took them to be in the uncontrolled category, and if they had type 2 diabetes, we took them to be in the controlled category, as these were the most populous categories in the training data.[6] For all other comorbidities/treatments, a missing value was taken to indicate absence of that comorbidity/treatment.

Reliable ethnicity data were not available, and all individuals were assigned to "White British". In the 2011 Scottish census, 96% of the population was in this ethnic group.[10] The most fine-grained residential location information available in our dataset was data zone, which is a geographical designation comprising of groups of UK Census output areas. Output areas typically consist of ~300 people, whereas data zones typically consist of 500–1,000 people.[11] Townsend Deprivation Scores [12] for each output area were obtained from the 2011 UK census.[11] We took the median value of Townsend Deprivation Scores for the

For peer review only - http://bmjopen.bmj.com/site/about/guidelines.xhtml

output areas comprising each data zone to get a deprivation score for each data zone. Missing values for Townsend Deprivation Scores were replaced with the mean value for the cohort. Missing values in the housing category variable were taken to indicate the individual was neither homeless, nor resident in a care home.

We used single imputation by chained equations [12] to impute missing values for body mass index (BMI). There is some evidence of an association between lower levels of socioeconomic status and higher BMI in economically developed countries.[13] Sex is known to be associated with BMI, as is coronary artery disease and diabetes.[14]

There were no missing values for any of the other independent variables.

Model validation

To calculate the risk for those who were unvaccinated, the baseline survival rate was provided on the following days: 0, 30, 60, 90, 183, 210, 240. We linearly interpolated the logarithm of the baseline survival rate to obtain values for intermediate days. We calculated probability of event for an individual either on the day of event, or the day of censoring.

We applied versions 2 and 3 of the QCovid algorithm to a random 10% sample of our cohort, and computed Harrell's Concordance,[15] the Brier score, Royston's D,[16] R^2 [17] and observed-expected (OE) ratio for the period 8 December 2020 to 15 June 2021. As well as calculating these metrics for the entire cohort sample, we also calculated them for several sub-cohorts. These sub-cohorts consisted of unvaccinated individuals, individuals followed up after first and second dose, and the age ranges, 19-64, 65-79 and 80-100.

Harrell's Concordance is a performance metric that characterises the tendency for people with higher hazard rates to have earlier events. It takes values between 0 and 1, with 0 indicating poorer performance and 1 indicating better performance. The Brier score is a measure of forecast accuracy that is equal to the mean squared prediction error in the case of a binary outcome variable. It takes values greater than 0, with zero indicating the best performance. Royston's D is a measure of 'separation' between survival curves. Higher values of Royston's D indicate predicted hazard rates have more discriminative ability. R^2 is a measure of the proportion of variation in survival time explained by the model. It takes values between 0 and 1, with 0 indicating poorer performance and 1 indicating better

For peer review only

performance. We calculated R^2 for survival models as defined in [17]. The OE ratio is the number of observed events divided by the expected number of events predicted by the model. A value of 1 indicates that the total number of events is exactly correctly predicted, and a value less than 1 indicates that the total number of events predicted was greater than observed. We made plots of observed vs expected risk on the day of event/censoring by vigintiles (20 groups) of predicted hazard rate. We also calculated recalibrated risk by scaling predicted risks by a multiplicative constant so that expected total number of events predicted was equal to observed total number of events. We used this recalibrated risk to calculate recalibrated Brier scores and made re-calibrated observed versus expected plots.

Reporting

This study is reported in accordance with the Transparent reporting of a multivariable prediction model for individual prognosis or diagnosis (TRIPOD) guidelines. [18]

Patient and public involvement

The EAVE II Public Advisory Group reviewed the online QCovid tools and the preliminary results from this study. Although overall in favour of this work, they expressed some concern at the lack of ethnicity data for our results, and that the online tools would need significant editing to be accessible to the publics.

Ethics and permissions

Ethical permission for this study was granted by the South East Scotland Research Ethics Committee 02 [12/SS/0201]. The Public Benefit and Privacy Panel Committee of Public Health Scotland, approved the linkage and analysis of the de–identified datasets for this project [1920-0279]. As we had approvals to use anonymised patient data without consent, there was no pathway for patients to opt out.

RESULTS

In our 10% cohort sample, there were 763 COVID-19 hospitalisations in the validation period, and 393 COVID-19 deaths. Table 1 shows the OE ratio, Harrell's C, Brier score, Royston's D, R^2 and recalibrated Brier score with outcomes of COVID-19 hospitalisation and COVID-19 death for the entire cohort sample, as well as subgroups stratified by follow up after different vaccine doses. Table 2 shows the same statistics, stratified by age group. Figure 1 plots the observed and expected risk of hospitalisation and death by vigintiles of the

For peer review only

245 hazard rate (HR) for the entire sample, the subset who were unvaccinated and those during
246 follow up after first and second dose, and Figure 2 shows the same set of figures but
247 recalibrated with the actual number of events. The vigintiles were numbered so that higher
248 vigintiles had a higher hazard rate (i.e., those in vigintile 1 were in the lowest hazard rate
249 category, those in vigintile 20 were in the higher hazard rate category).
250
251 **Table 1.** Performance metrics (with 95% confidence intervals) stratified by vaccination status
252 for COVID-19 hospitalisation and death

Group	Observed-expected ratio	C	Brier Score	Royston's D	Recalibrated Brier Score	R^2
**Hospitalisation**						
Everyone	0.24	0.84 (0.82 - 0.86)	0.0009 (0.00084 - 0.00096)	2.12 (2.022 - 2.218)	0.00091 (0.00085 - 0.00097)	0.52 (0.49 - 0.54)
Unvaccinated	0.23	0.73 (0.71 - 0.75)	0.00209 (0.00193 - 0.00225)	1.33 (1.2124 - 1.4476)	0.00211 (0.00195 - 0.00227)	0.3 (0.26 - 0.33)
First and second dose vaccinated	0.33	0.72 (0.66 - 0.78)	0.00015 (0.00013 - 0.00017)	1.43 (1.136 - 1.724)	0.00015 (0.00013 - 0.00017)	0.33 (0.23 - 0.42)
**Death**						
Total population	0.26	0.92 (0.9 - 0.94)	0.00036 (0.00032 – 0.004)	2.77 (2.6132 - 2.9268)	0.00036 (0.00032 – 0.0004)	0.65 (0.62 - 0.67)

For peer review only

Unvaccinated	0.34	0.88 (0.86 - 0.9)	0.00077 (0.00069 - 0.00085)	2.35 (2.1736 - 2.5264)	0.00077 (0.00069 - 0.00085)	0.57 (0.53 - 0.6)
First and second dose vaccinated	0.13	0.94 (0.92 - 0.96)	0.00001 (0.00008 - 0.00012)	3.09 (2.7372 - 3.4428)	1.0001 (0.00008 - 0.00012)	0.7 (0.64 - 0.74)

Table 2. Performance metrics (with 95% confidence intervals) for COVID-19 hospitalisation and death stratified by age group

Age Group	Observed -expected ratio	C	Brier Score	Royston's D	Recalibrated Brier Score	R^2
**Hospitalisation**						
Age 18-64	0.21	0.82 (0.8 - 0.84)	0.00067 (0.00061 - 0.00073)	1.9 (1.7628 - 2.0372)	0.00068 (0.00062 - 0.00074)	0.46 (0.42 - 0.5)
Age 65-79	0.29	0.80 (0.78 - 0.82)	0.00112 (0.00098 - 0.00126)	1.70 (1.504 - 1.896)	0.00113 (0.00099 - 0.00127)	0.41 (0.35 - 0.46)
Age 80+	0.27	0.78 (0.76 - 0.8)	0.00255 (0.0022 - 0.0029)	1.55 (1.3344 - 1.7656)	0.00258 (0.00223 - 0.00293)	0.36 (0.3 - 0.43)
**Death**						
Age 18-64	0.15	0.85 (0.81 - 0.89)	0.00007 (0.00005 – 0.00009)	1.96 (1.5288 - 2.3912)	0.00007 (0.00005 – 0.00009)	0.48 (0.36 - 0.58)

For peer review only

Age 65-79	0.28	0.82 (0.8 - 0.84)	0.00052 (0.00042 - 0.00062)	1.77 (1.476 - 2.064)	0.00052 (0.00042 - 0.00062)	0.43 (0.34 - 0.51)
Age 80+	0.3	0.73 (0.71 - 0.75)	0.00309 (0.0027 - 0.00348)	1.23 (1.034 - 1.426)	0.00311 (0.0027 - 0.00352)	0.26 (0.2 - 0.33)

Vaccine dose

Focusing firstly on hospitalisations stratified by vaccine dose (shown in the top half of Table 1), according to Harrell's C, Royston's D and the R^2 metric, performance was similar across all sub-groups, but better for the entire sample cohort. This is because combining data for unvaccinated with vaccinated people created a large number of additional concordant pairs. Both the Brier score and the recalibrated Brier scores were noticeably worse for the unvaccinated than for those who had one or two vaccine doses, or the entire sample cohort.

In terms of COVID-19 deaths (shown in the bottom half of Table 1), we found that the model performed similarly according to Harrell's C across the vaccine dose follow-up categories. Again, across the other metrics bar the OE ratio, performance was worse for the unvaccinated than for those who had one or two vaccine doses, or for the entire sample cohort.

For both events, the performance according to Harrell's C was very good, and was similar to that in the original study on their validation cohort (0.84 (0.82, 0.86) for hospitalisation in this analysis vs 0.85 in the original study, and 0.92 (0.90 – 0.94) in this analysis vs 0.93 in the original study) [6]. The D statistic performance in this analysis was worse than in the original study (2.21 (2.022, 2.218) vs 2.83 (2.59, 3.08) for hospitalisation and 2.77 (2.61, 2.93) vs 3.46 (3.19, 3.73) for death).

Age

Looking at the results for COVID-19 hospitalisation stratified by age (shown in top half of Table 2), the model performed less well for the older age groups (65 – 79, ≥80) compared to

For peer review only

the younger group (18 – 64) according to all metrics, except for the OE ratio. This pattern is also repeated for COVID-19 death stratified by age (shown in the bottom half of Table 2).

Vigintile plots

Figure 1 shows a set of vigintile plots comparing the predicting risk to the observed risk for the full cohort and two sub cohorts – those who were unvaccinated and those with a first and second dose. Figure 2 shows the same set of vigintile plots, but with the risks recalibrated.

The vigintile plots on the left-hand side of Figure 1 show the model's performance in terms of predicting COVID-19 hospitalisations (left hand side) and deaths (right hand size). For both events, the models tended to overestimate the events across all stratifications. The estimates tended to be worse for the higher risk vigintiles.

Figure 2 shows the same plots after we recalibrated the models. After this we found that most of the vigintiles had a good estimation of the number of events after recalibration. Across the stratifications, there was no consistent pattern of over-estimating or underestimating the number of events for specific vigintiles. The predictions for the ≥80 years sub-cohort, particularly for death, were not as good as for the other cohorts. This may have been due to the small sample size combined with the larger risk scores.

DISCUSSION

Statement of principal findings

In this external validation of the QCovid 2 and 3 algorithms in Scotland, we found the algorithms performed well with regards to discrimination as measured by the C statistic. The total number of events was overestimated for hospitalisations and deaths across all subgroups. Performance was broadly similar to that in the original study.[6]

Strengths and limitations

Our study had a number of important strengths. As in our previous study, [4] we developed a unique linked dataset covering 99% of the population resident in Scotland. The EAVE II database [19] is one of the few national individual patient-level linked research databases in the world.[20] We evaluated the performance of the QCovid 2 and 3 algorithms according to all metrics used in the original study [6] and in the common protocol agreed between the four UK nations.[8]

For peer review only

However, our work has several limitations. Firstly, we did not have access to ethnicity data, so all individuals were set to 'White British' ethnicity. We believe modal substitution for ethnicity was reasonable because the most recent Scottish census indicated that 96% of the residents of Scotland identified their ethnicity as 'White'.[10] There was significant missingness in the BMI data, with 2,495,504 (55.6%) missing values. We used single imputation by chained equations to impute these missing values for BMI. Multiple imputation was not used due to limitations on computational resources. The most fine-grained residential location information available in our dataset was data zone, which typically consists of multiple 2011 UK census output areas. We took the median value of the Townsend Deprivation Scores for the output areas comprising each data zone to get a deprivation score for each data zone. Missing values of Townsend Deprivation Scores were replaced with the average value for the cohort. Higher levels of deprivation as measured by Townsend Deprivation Scores was associated with increased predicted risk of COVID-19 hospitalisation and death in the QCovid 2 and 3 algorithms. We also did not have data for several clinical risk variables, so individuals were assigned to the category for absence of the condition. This will have had the effect of under-estimating risk in people with these characteristics. We also validated on a 10% sample randomly chosen rather than the full EAVE II cohort due to limitations on computational resources. This led to there being few samples in some of the higher risk groups, and wider confidence intervals on the metrics.

Interpretation

The QCovid 2 and 3 risk prediction algorithms performed well in the Scottish population in the period they were trained for.

Implications for policy, practise and research

Our results indicate that QCovid 2 and 3 would have been appropriate for use as a risk prediction tool for COVID-19 hospitalisation and death in Scotland during our study period. For future research, taking UK-wide perspectives on data availability when developing risk prediction tools should be considered if these tools are to be used nationally.

CONCLUSION

For peer review only

Risk prediction tools are valuable for identifying individuals at the highest risk of experiencing severe outcomes and can be used by policy makers to help mitigate these risks. However, these tools must be externally validated on cohorts that were not used to train them. We evaluated the QCovid2 and 3 algorithms in Scotland using a variety of metrics and across several sub-groups. We found good performance overall, and many measures were similar to those in the original study.

Acknowledgements: The authors would like to thank staff at Public Health Scotland, Albasoft Ltd, the general practices that contributed data to the study and the EAVE II Collaborators. AS, JM and CR serve on The Scottish Government's COVID-19 Chief Medical Officer's Advisory Group and the New and Emerging Respiratory Virus Threats Advisory Group (NERVTAG) Risk Stratification Subgroup.

Data availability statement: All code, metadata and documentation for this project is publicly available at https://github.com/EAVE-II/QCovid2-3-validation. A data dictionary is available at https://github.com/EAVE-II/EAVE-II-data-dictionary. Most of the data that were used in this study are highly sensitive and will not be made available publicly.

Contributors: AS and JHC conceptualised the study. SK carried out the formal analysis. TM wrote an initial draft of the manuscript. All authors assisted with review and editing. CR and TM have verified the underlying data.

Competing interests: Dr. Hippisley-Cox reports grants from MRC, grants from Wellcome Trust, grants from NIHR, during the conduct of the study; JHC is a founder and shareholder of ClinRisk ltd and was its medical director until 31st May 2019. ClinRisk Ltd produces open and closed source software to implement clinical risk algorithms (outside this work) into clinical computer systems. JHC was chair of the NERVTAG risk stratification subgroup and a member of SAGE COVID-19 groups and the NHS group advising on prioritisation of use of monoclonal antibodies in COVID-19 infection. Dr. Sheikh reports grants from NIHR, grants from MRC, grants from HDR UK, during the conduct of the study. All other authors report no conflict of interest.

Funding: National Institute for Health Research (NIHR) following a commission by the Chief Medical Officer for England. EAVE II is funded by the Medical Research Council (MC_PC_19075) with the support of BREATHE: the Health Data Research Hub for Respiratory Health (MC_PC_19004), which is funded through the UK Research and Innovation Industrial Strategy Challenge Fund and delivered through Health Data Research

For peer review only

UK. Additional support has been provided through Public Health Scotland and the Community Health and Social Care Directorate of the Scottish Government.

References

1. *Deaths involving coronavirus (COVID-19) in Scotland*. 2023; Available from: https://www.nrscotland.gov.uk/statistics-and-data/statistics/statistics-by-theme/vital-events/general-publications/weekly-and-monthly-data-on-births-and-deaths/deaths-involving-coronavirus-covid-19-in-scotland
2. *New technology to help identify those at high risk from COVID-19* [cited 2023; Available from: https://www.gov.uk/government/news/new-technology-to-help-identify-those-at-high-risk-from-covid-19.
3. Nafilyan, V., et al., *An external validation of the QCovid risk prediction algorithm for risk of mortality from COVID-19 in adults: a national validation cohort study in England.* The Lancet Digital Health, 2021. **3**(7): p. e425-e433.
4. Simpson, C.R., et al., *External validation of the QCovid risk prediction algorithm for risk of COVID-19 hospitalisation and mortality in adults: national validation cohort study in Scotland.* Thorax, 2022. **77**(5): p. 497-504.
5. Lyons, J., et al., *Validating the QCOVID risk prediction algorithm for risk of mortality from COVID-19 in the adult population in Wales, UK.* Int J Popul Data Sci, 2020. **5**(4): p. 1697.
6. Hippisley-Cox, J., et al., *Risk prediction of covid-19 related death and hospital admission in adults after covid-19 vaccination: national prospective cohort study.* BMJ, 2021. **374**: p. n2244.
7. Hippisley-Cox, J., D. Stables, and M. Pringle, *QRESEARCH: a new general practice database for research.* Inform Prim Care, 2004. **12**(1): p. 49-50.
8. Kerr, S., et al., *Common protocol for validation of the QCOVID algorithm across the four UK nations.* BMJ Open, 2022. **12**(6): p. e050994.
9. *EAVE II Data Dictionary*, E.I. Analysts, Editor. 2021.
10. *Scotland's Census - Ethnicity*. 2021; Available from: https://www.scotlandscensus.gov.uk/census-results/at-a-glance/ethnicity/.
11. Service., O.f.N.S.N.R.o.S.N.I.S.a.R.A.U.D. *2011 UK Census Townsend Deprivation Scores*. 2011 [cited 2023; Available from: https://statistics.ukdataservice.ac.uk/dataset/2011-uk-townsend-deprivation-scores.
12. Little, R.J. and D.B. Rubin, *Single imputation methods.* Statistical analysis with missing data, 2002: p. 59-74.
13. McLaren, L., *Socioeconomic status and obesity.* Epidemiologic reviews, 2007. **29**(1): p. 29-48.
14. Bays, H.E., R.H. Chapman, and S. Grandy, *The relationship of body mass index to diabetes mellitus, hypertension and dyslipidaemia: comparison of data from two national surveys.* Int J Clin Pract, 2007. **61**(5): p. 737-47.
15. Harrell, F.E., Jr., et al., *Evaluating the yield of medical tests.* Jama, 1982. **247**(18): p. 2543-6.
16. Royston, P. and W. Sauerbrei, *A new measure of prognostic separation in survival data.* Stat Med, 2004. **23**(5): p. 723-48.

For peer review only - http://bmjopen.bmj.com/site/about/guidelines.xhtml

17. Royston, P., *Explained variation for survival models.* The Stata Journal, 2006. **6**(1): p. 83-96.
18. Moons, K.G., et al., *Transparent Reporting of a multivariable prediction model for Individual Prognosis or Diagnosis (TRIPOD): explanation and elaboration.* Ann Intern Med, 2015. **162**(1): p. W1-73.
19. Simpson, C.R., et al., *Early Pandemic Evaluation and Enhanced Surveillance of COVID-19 (EAVE II): protocol for an observational study using linked Scottish national data.* BMJ Open, 2020. **10**(6): p. e039097.
20. Simpson, C.R., et al., *The UK's pandemic influenza research portfolio: a model for future research on emerging infections.* Lancet Infect Dis, 2019. **19**(8): p. e295-e300.

FIGURE TITLES

Figure 1. Vigintile plots of expected vs observed risks for the cohort and a selection of sub-cohorts

Figure 2. Vigintile plots of expected vs observed recalibrated risks for the cohort and a selection of sub-cohorts

For peer review only

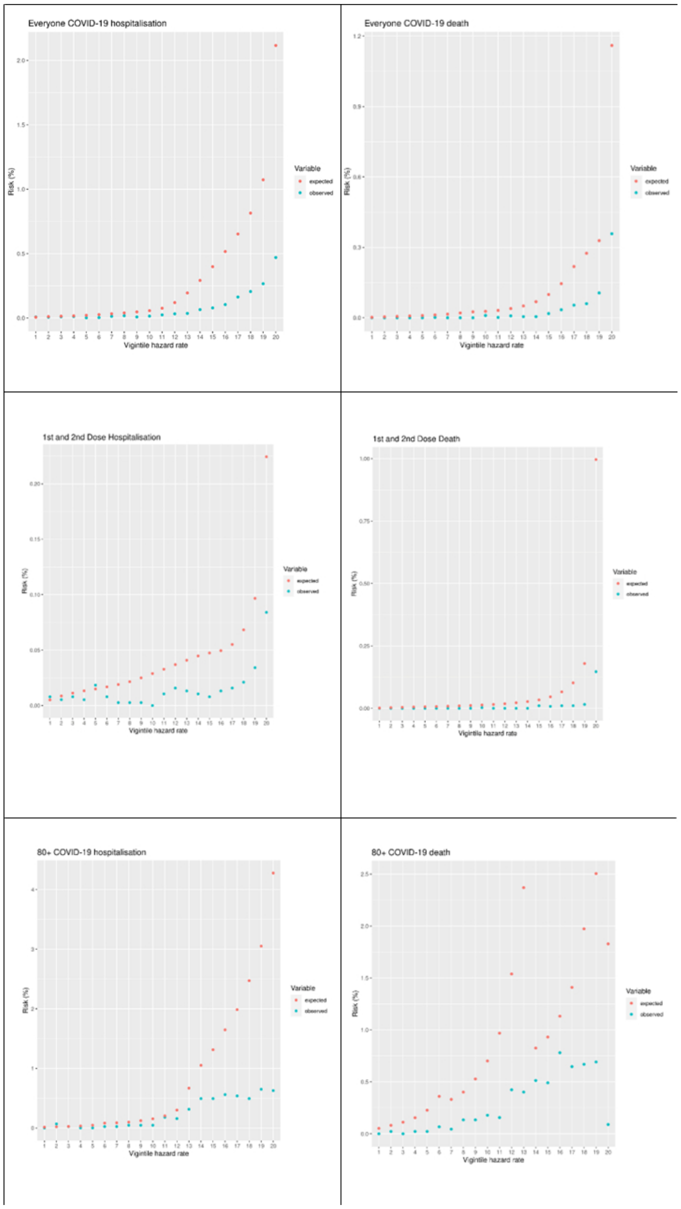

Vigintile plots of expected vs observed risks for the cohort and a selection of sub-cohorts.

481x855mm (38 x 38 DPI)

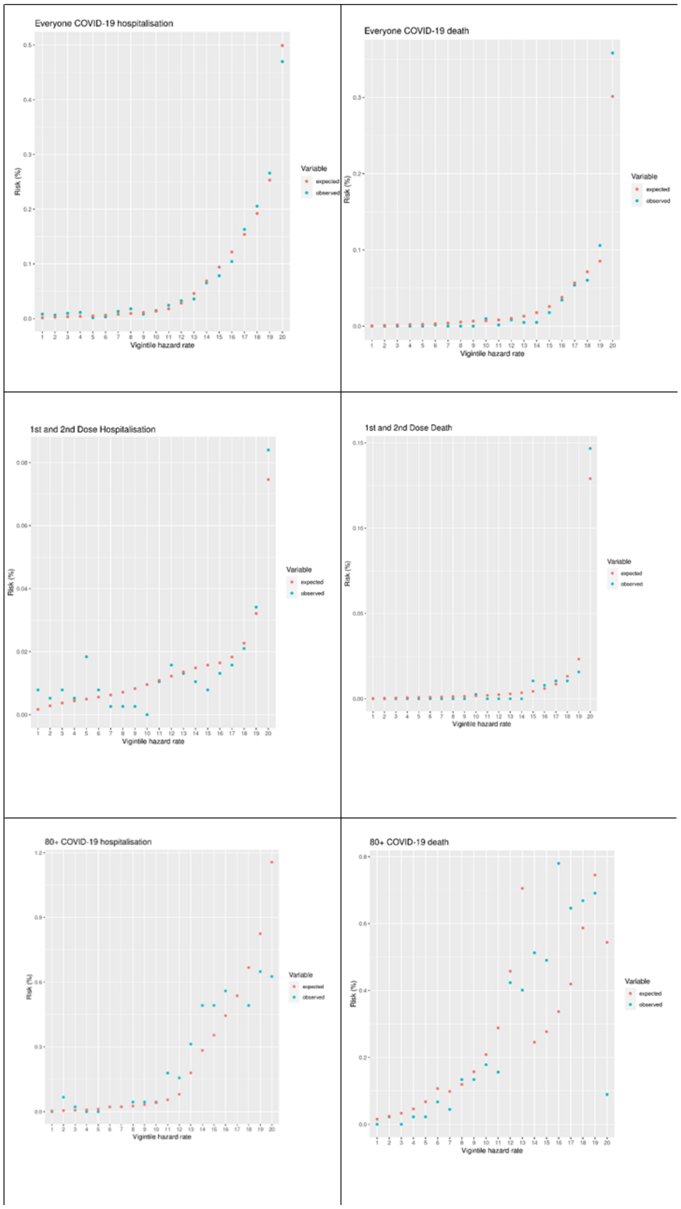

Vigintile plots of expected vs observed recalibrated risks for the cohort and a selection of sub-cohorts.

481x855mm (38 x 38 DPI)

Table S1: Summary statistics for the full cohort and 10% sample

Characteristics	Levels	Entire cohort	10% cohort sample
Total		4650585 (100.0)	465058 (100.0)
COVID-19 Deaths		4049 (0.1)	393 (0.1)
COVID-19 Hospitalisations		9547 (0.2)	763 (0.2)
Sex	Female	2358990 (50.7)	235821 (50.7)
	Male	2291595 (49.3)	229237 (49.3)
Age	Mean (SD)	50.3 (18.8)	50.3 (18.9)
Age group	19-39	1581219 (34.0)	158052 (34.0)
	40-59	1533605 (33.0)	152985 (32.9)
	60-74	978827 (21.0)	98190 (21.1)
	75-100	556934 (12.0)	55831 (12.0)
SIMD quintile	1 - Most deprived	925925 (19.9)	92546 (19.9)
	2	918884 (19.8)	92073 (19.8)
	3	918091 (19.7)	91868 (19.8)
	4	921838 (19.8)	92219 (19.8)
	5 - Least deprived	924963 (19.9)	92392 (19.9)
	(Missing)	40884 (0.9)	3960 (0.9)
Urban rural classification	Large Urban Areas	1671719 (35.9)	166887 (35.9)
	Other Urban Areas	1652152 (35.5)	165274 (35.5)
	Accessible Small Towns	424031 (9.1)	42739 (9.2)
	Remote Small Towns	221513 (4.8)	22271 (4.8)
	Accessible Rural	413436 (8.9)	41170 (8.9)
	Remote Rural	226850 (4.9)	22757 (4.9)
	(Missing)	40884 (0.9)	3960 (0.9)
Ethnicity	Asian	102490 (2.2)	10250 (2.2)
	Black	25123 (0.5)	2567 (0.6)
	Mixed	21139 (0.5)	2148 (0.5)

For peer review only

	Other	23584 (0.5)	2383 (0.5)
	Unknown	1562888 (33.6)	156408 (33.6)
	White	2915361 (62.7)	291302 (62.6)
Number of risk groups	0	2725853 (58.6)	272930 (58.7)
	1	1249848 (26.9)	125016 (26.9)
	2	434728 (9.3)	43153 (9.3)
	3 - 4	210691 (4.5)	21024 (4.5)
	5+	29465 (0.6)	2935 (0.6)
BMI	15-18.5	48651 (1.0)	4772 (1.0)
	18.5-25	543001 (11.7)	54377 (11.7)
	25-30	674760 (14.5)	67921 (14.6)
	30-35	431788 (9.3)	43170 (9.3)
	35-40	198273 (4.3)	19835 (4.3)
	40+	133917 (2.9)	13226 (2.8)
	(Missing)	2620195 (56.3)	261757 (56.3)
Housing category	Neither	4624584 (99.4)	462533 (99.5)
	Care home	25217 (0.5)	2457 (0.5)
	Homeless	784 (0.0)	68 (0.0)
Learning disability/Down's syndrome	Neither	4600228 (98.9)	460016 (98.9)
	Down's syndrome	1526 (0.0)	144 (0.0)
	Learning disability	48831 (1.0)	4898 (1.1)
Chronic Kidney Disease	No CKD	4484131 (96.4)	448600 (96.5)
	CKD 3	166431 (3.6)	16455 (3.5)
	CKD 4	19 (0.0)	*
	CKD 5	4 (0.0)	*
Atrial Fibrillation		114239 (2.5)	11502 (2.5)
Asthma		494549 (10.6)	49499 (10.6)
Blood cancer		20162 (0.4)	2049 (0.4)

For peer review only

Congestive Cardiac Failure		46961 (1.0)	4748 (1.0)
Cerebral Palsy		5521 (0.1)	529 (0.1)
Coronary heart disease		191035 (4.1)	19132 (4.1)
Liver cirrhosis		10882 (0.2)	1116 (0.2)
Congenital heart disease		18969 (0.4)	1854 (0.4)
COPD		118884 (2.6)	11922 (2.6)
Dementia		23258 (0.5)	2202 (0.5)
Diabetes Type 1		65238 (1.4)	6434 (1.4)
Diabetes Type 2		205608 (4.4)	20446 (4.4)
Epilepsy		4032 (0.1)	386 (0.1)
Hip, wrist, spine, humerus fracture		1638 (0.0)	140 (0.0)
HIV/AIDS		18937 (0.4)	1865 (0.4)
Severe combined immunodeficiency syndrome		9707 (0.2)	987 (0.2)
Neurological conditions		6916 (0.1)	668 (0.1)
Parkinsons disease		23496 (0.5)	2367 (0.5)
Pulmonary hypertension		11937 (0.3)	1212 (0.3)
Cystic fibrosis, bronchiectasis or alveolitis		88090 (1.9)	8852 (1.9)
Peripheral vascular disease		5452 (0.1)	538 (0.1)
SLE or rheumatoid arthritis		19750 (0.4)	1892 (0.4)
Lung, oral cancer		205 (0.0)	8 (0.0)

For peer review only

Severe mental illness		115619 (2.5)	11431 (2.5)
Sickle cell disease or combined immune deficiency syndrome		41948 (0.9)	4198 (0.9)
Stroke, transient ischaemic attack		22733 (0.5)	2309 (0.5)
Venous thromboembolism		265285 (5.7)	26697 (5.7)

For peer review only

Box S1: Predictor variables in the QCovid 3 algorithm

• Vaccine dose
• Age in years (continuous)
• Sex
• Ethnicity in ten categories (Bangladeshi, Black African, Black Caribbean, Chinese, Indian, Mixed, Pakistani, White British, White other, Other, unknown)
• Townsend deprivation score (continuous)
• Body Mass Index (kg/m2)
• Accommodation (Neither homeless nor care home, care home or nursing home)
• Chronic kidney disease (CKD) – (no CKD, CKD3, CKD4, CKD5, unknown)
• Learning disability (No learning disability, Down's Syndrome, other learning disability)
• Chemotherapy in last 12 months (none, Chemotherapy group A, B, C, unknown)
• Blood Cancer
• Bone Marrow transplant in the past 6 months
• Rare Lung Diseases (cystic fibrosis, bronchiectasis, or alveolitis)
• Respiratory cancer
• Radiotherapy in last 6 months
• Solid organ transplant
• Prescribed immunosuppressant medication by GP
• Prescribed leukotriene or long-acting beta blockers
• Prescribed regular prednisolone
• Sickle cell disease
• Diabetes
• Chronic obstructive pulmonary disease (COPD)
• Asthma
• Rare pulmonary diseases
• Pulmonary hypertension or pulmonary fibrosis
• Coronary heart disease
• Stroke
• Atrial Fibrillation
• Congestive cardiac failure
• Venous thromboembolism
• Peripheral vascular disease
• Congenital heart disease
• Dementia
• Heart Failure
• Parkinson's disease
• Epilepsy
• Rare neurological conditions
• Cerebral palsy
• Severe mental illness (bipolar disorder, schizophrenia, severe depression)
• Osteoporotic fracture
• Rheumatoid arthritis or Systemic lupus erythematosus
• Cirrhosis of the liver
• Inflammatory Bowel Disease
• HIV/AIDS
• Severe Combined Immunodeficiency
• Previous Positive SARS-CoV-2 positive test result
• Background rate of RT-PCR positivity per 100,000 people

For peer review only

Reporting checklist for prediction model development/validation.

Based on the TRIPOD guidelines.

Instructions to authors

Complete this checklist by entering the page numbers from your manuscript where readers will find each of the items listed below.

Your article may not currently address all the items on the checklist. Please modify your text to include the missing information. If you are certain that an item does not apply, please write "n/a" and provide a short explanation.

Upload your completed checklist as an extra file when you submit to a journal.

In your methods section, say that you used the TRIPOD reporting guidelines, and cite them as:

Collins GS, Reitsma JB, Altman DG, Moons KG. Transparent reporting of a multivariable prediction model for individual prognosis or diagnosis (TRIPOD): The TRIPOD statement.

For peer review only

		Reporting Item	Page Number
**Title**			
	#1	Identify the study as developing and / or validating a multivariable prediction model, the target population, and the outcome to be predicted.	1
**Abstract**			

	#2	Provide a summary of objectives, study design, setting, participants, sample size, predictors, outcome, statistical analysis, results, and conclusions.	3
**Introduction**			
	#3a	Explain the medical context (including whether diagnostic or prognostic) and rationale for developing or validating the multivariable prediction model, including references to existing models.	3
	#3b	Specify the objectives, including whether the study describes the development or validation of the model or both.	4
**Methods**			
Source of data	#4a	Describe the study design or source of data (e.g., randomized trial, cohort, or registry data), separately for the development and validation data sets, if applicable.	4
Source of data	#4b	Specify the key study dates, including start of accrual; end of accrual; and, if applicable, end of follow-up.	4,6
Participants	#5a	Specify key elements of the study setting (e.g., primary care, secondary care, general	4

For peer review only

		population) including number and location of centres.	
Participants	#5b	Describe eligibility criteria for participants.	4
Participants	#5c	Give details of treatments received, if relevant	NA
Outcome	#6a	Clearly define the outcome that is predicted by the prediction model, including how and when assessed.	6
Outcome	#6b	Report any actions to blind assessment of the outcome to be predicted.	No actions necessary
Predictors	#7a	Clearly define all predictors used in developing or validating the multivariable prediction model, including how and when they were measured	Supplementary material 4
Predictors	#7b	Report any actions to blind assessment of predictors for the outcome and other predictors.	No actions necessary
Sample size	#8	Explain how the study size was arrived at.	6
Missing data	#9	Describe how missing data were handled (e.g., complete-case analysis, single imputation, multiple imputation) with details of any imputation method.	5

For peer review only

Statistical analysis methods	#10a	If you are developing a prediction model describe how predictors were handled in the analyses.	NA – not developing a model
Statistical analysis methods	#10b	If you are developing a prediction model, specify type of model, all model-building procedures (including any predictor selection), and method for internal validation.	NA – not developing a model
Statistical analysis methods	#10c	If you are validating a prediction model, describe how the predictions were calculated.	6
Statistical analysis methods	#10d	Specify all measures used to assess model performance and, if relevant, to compare multiple models.	6
Statistical analysis methods	#10e	If you are validating a prediction model, describe any model updating (e.g., recalibration) arising from the validation, if done	7
Risk groups	#11	Provide details on how risk groups were created, if done.	5
Development vs. validation	#12	For validation, identify any differences from the development data in setting, eligibility criteria, outcome, and predictors.	3,4

For peer review only

Results

Participants	#13a	Describe the flow of participants through the study, including the number of participants with and without the outcome and, if applicable, a summary of the follow-up time. A diagram may be helpful.	
Participants	#13b	Describe the characteristics of the participants (basic demographics, clinical features, available predictors), including the number of participants with missing data for predictors and outcome.	Supplementary material p1
Participants	#13c	For validation, show a comparison with the development data of the distribution of important variables (demographics, predictors and outcome).	NA – we are validating an external algorithm trained on external data we do not have access to
Model development	#14a	If developing a model, specify the number of participants and outcome events in each analysis.	NA – not developing a model
Model development	#14b	If developing a model, report the unadjusted association, if calculated between each candidate predictor and outcome.	NA – not developing a model
Model specification	#15a	If developing a model, present the full prediction model to allow predictions for	NA – not developing a model

For peer review only

		individuals (i.e., all regression coefficients, and model intercept or baseline survival at a given time point).	
Model specification	#15b	If developing a prediction model, explain how to the use it.	NA – not developing a model
Model performance	#16	Report performance measures (with CIs) for the prediction model.	8,9,10
Model-updating	#17	If validating a model, report the results from any model updating, if done (i.e., model specification, model performance).	8,9,10
**Discussion**			
Limitations	#18	Discuss any limitations of the study (such as nonrepresentative sample, few events per predictor, missing data).	11,12
Interpretation	#19a	For validation, discuss the results with reference to performance in the development data, and any other validation data	12
Interpretation	#19b	Give an overall interpretation of the results, considering objectives, limitations, results from similar studies, and other relevant evidence.	12

For peer review only

Implications	#20	Discuss the potential clinical use of the model and implications for future research	12
**Other information**			
Supplementary information	#21	Provide information about the availability of supplementary resources, such as study protocol, Web calculator, and data sets.	13
Funding	#22	Give the source of funding and the role of the funders for the present study.	14

None The TRIPOD checklist is distributed under the terms of the Creative Commons Attribution License CC-BY. This checklist can be completed online using https://www.goodreports.org/, a tool made by the EQUATOR Network in collaboration with Penelope.ai

For peer review only
